# Antibiotic-sterol interactions provide insight into the selectivity of natural aromatic analogues of amphotericin B and their photoisomers

**DOI:** 10.1038/s41598-023-28036-x

**Published:** 2023-01-14

**Authors:** Julia Borzyszkowska-Bukowska, Jacek Czub, Paweł Szczeblewski, Tomasz Laskowski

**Affiliations:** 1 Department of Pharmaceutical Technology and Biochemistry and BioTechMed Centre, Faculty of Chemistry, Gdańsk University of Technology, Gabriela Narutowicza Str. 11/12, 80-233 Gdańsk, Poland; 2 Department of Physical Chemistry and BioTechMed Centre, Faculty of Chemistry, Gdańsk University of Technology, Gabriela Narutowicza Str. 11/12, 80-233 Gdańsk, Poland

**Keywords:** Computational biophysics, Computational chemistry, Computational chemistry, Computational chemistry

## Abstract

Aromatic heptaene macrolides (AHMs) belong to the group of polyene macrolide antifungal antibiotics. Members of this group were the first to be used in the treatment of systemic fungal infections. Amphotericin B (AmB), a non-aromatic representative of heptaene macrolides, is of significant clinical importance in the treatment of internal mycoses. It includes the all-trans heptaene chromophore, whereas the native AHMs contain two cis-type (Z) double bonds within the chromophore system. Lately we have proven that it is possible to obtain AHMs’ stable derivatives in the form of all-trans (AmB-type) isomers by photochemical isomerization. Our further studies have shown that such alteration leads to the improvement of their selective toxicity in vitro. Computational experiments carried out so far were only an initial contribution in the investigation of the molecular basis of the mechanism of action of AHMs and did not provide explanation to observed differences in biological activity between the native (cis-trans) and isomeric (all-trans) AHMs. Herein, we presented the results of two-dimensional metadynamics studies upon AmB and its aromatic analogues (AHMs), regarding preferable binary antibiotic/sterol complexes orientation, as well as more detailed research on the behaviour of AHMs’ alkyl-aromatic side chain in cholesterol- or ergosterol-enriched lipid bilayers.

## Introduction

Polyene macrolides are the first known antibiotics ever used in the treatment of fungal infections^1,2^. This group of antibiotics exhibit all features essential for the effective antifungal drugs, namely: high antifungal activity, broad antifungal spectrum, fungicidal mode of action, inability to induce resistant strains and even overcoming the multidrug resistance (MDR) of fungi^3^. The latter qualities are crucial in the light of the emerging crisis of antibiotic-resistant pathogens^4,5^. Until today, polyene macrolides are still considered to be the most significant group of antimycotics.

All members of the macrolide group contain a large, 20–44-membered macrocyclic lactone (macrolactone ring) and, in most cases, one glycosidically bound monosaccharide moiety in their structure. Polyene macrolides are distinguished from the rest of the group due to the presence of a conjugated double-bond system, constituting the non-polar chromophore. Moreover, there is another specific fragment of a macrolactone ring, which is called ‘a polyol chain’ as it contains a number of polar groups such as carbonyls, hydroxyls and epoxides. The polar and non-polar fragments of a macrolide ring are located in direct opposition, which is a crucial structural feature as it results in amphiphilic nature of these compounds. Finally, depending on the number of the double bonds in a chromophore, polyene macrolides are classified as trienes, tetraenes, pentaenes, hexaenes, heptaenes and octaenes^6,7^.

**Figure 1:**
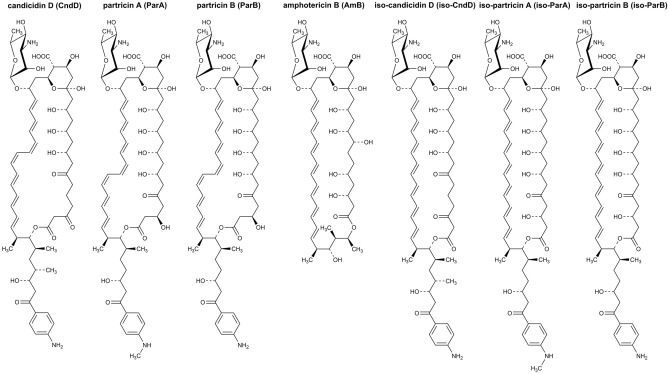
The structures of amphotericin B (AmB), native (cis-trans) aromatic heptaene macrolides (CndD, ParA, ParB) and their all-trans photoisomers (iso-CndD, iso-ParA, iso-ParB).

In the class of polyene macrolides with seven conjugated double bonds there is a subgroup of aromatic heptaene macrolides (AHMs)^8^. There are two main structural features, distinguishing non-aromatic heptaenes and aromatic heptaenes. Members of the AHM group are characterised by: 1) a presence of an aminoacetophenone moiety attached to an aliphatic side chain and 2) different, in comparison to non-aromatic heptaenes, geometry of the chromophore (cis-trans for AHM vs. all-trans for non-AHM). The most widely known representatives of AHMs are candicidin D (CndD, syn. levorin A2, ascosin A2, FR-008), partricin A (ParA, syn. gedamycin) and partricin B (ParB, syn. vacidin A), whereas amphotericin B (AmB) is regarded as an unquestionable leader of the non-AHM family (Fig. 1).

According to our recent studies on AHM, it has been unambiguously proven that it is possible to obtain a stable isomer of an antibiotic molecule with an altered polyene region^9,10^. Aromatic heptaenes can be transformed, via photochemical isomerisation, into its all-trans isomers with the chromophore geometry identical to the one of AmB. The resulting all-trans AHMs (iso-AHMs) exhibit more similar structure to AmB, yet still contain an aromatic side chain that surely has an effect on their interactions with mammalian and fungal sterols within the lipid bilayer environment.

Although polyene macrolides were studied for dozens of years, there is still much to be explained in this area. For instance, the molecular mechanism of action of polyene antibiotics is still not fully understood^11^. Such knowledge is a key element in development of suitable pharmacotherapy. For now, it is widely accepted that these antibiotics affect cell membranes^12^ and several modes of actions of these compounds toward a lipid bilayer have been proposed^13^. Most theories postulate that the basis for their selective fungicidal activity lies in the interaction of the antibiotics with the components of a lipid bilayer, in most cases sterol molecules, which vary among living organisms, i.e., mammals and fungi.

In case of aromatic heptaene macrolides, the postulated mode of action has been proposed on the basis of their structural resemblance to AmB. Preliminary biophysical studies indicated that AHMs indeed cause membrane disruption and ion leakage from a cell^14–16^, yet the question whether the lethal effect is a result of transmembrane channel formation (similarly to AmB^17^) still remains unanswered. Hence, while the molecular basis of biological activity of polyene macrolides in general remains not fully elucidated and still widely discussed in a worldwide scientific drug design community^18–21^, the mechanism of antifungal action of AHMs is a truly uncharted territory.

Previously, we have presented an initial, computational contribution to the description of the molecular mechanism of action of aromatic heptaene macrolides (AHMs) and their all-trans photoisomers^22^. Umbrella sampling calculations were carried out with the assumption that the sterols interact with AHMs in a canonical way, i.e., binding to the polyene part of the antibiotic molecules. Although the resulting differences in energetic description of native AHM/cholesterol and AHM/ergosterol interactions were in line with their established biological activity, the same could not be stated on the data obtained for the iso-AHMs, especially considering their in vitro selective toxicity. These results have suggested that the heptaene chromophore of aromatic polyene macrolides might not be a preferred site of AHM/sterol interactions, hence could not serve as a basis for a computational explanation of experimentally observed selective toxicity index (STI). Therefore, in this contribution we have applied extensive molecular dynamics studies with the use of 2D-metadynamics approach in order to establish the preferred relative orientation of three AHMs: candicidin D, partricin A and partricin B, as well as their isoforms, in interactions with cholesterol (Chol) and ergosterol (Erg). Moreover, we have also focused on the alkyl-aromatic side chain of AHMs and its role in the antibiotics’ dynamics and its interactions with the molecular targets. Finally, since similar calculations were actually never conducted for amphotericin B (AmB) and the preferred geometry of AmB/sterol interactions was established in one-dimensional computational experiments^23^, as a reference we have also examined single AmB molecules embedded in Chol- and Erg-containing lipid-bilayers using 2D-metadynamics approach.

## Results

### Free energy landscapes

**Figure 2:**
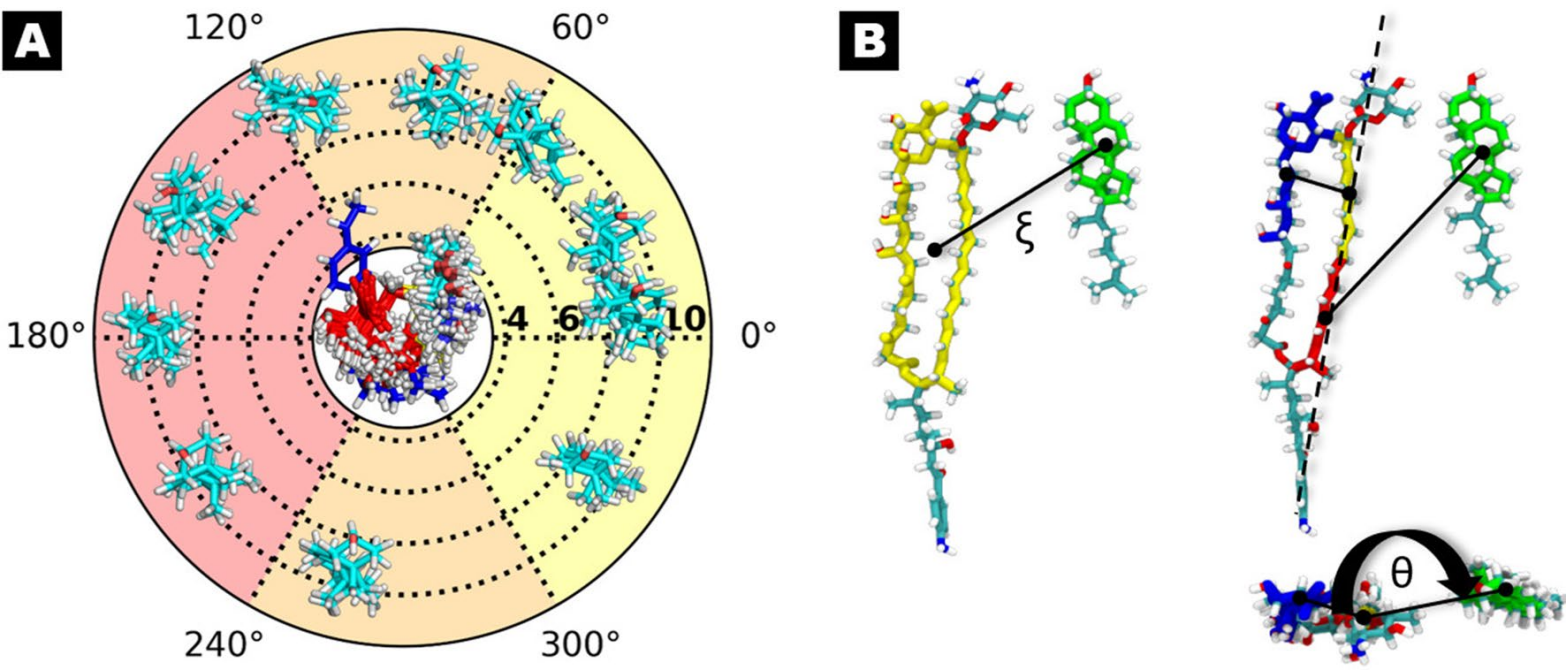
Panel (**A**): dihedral angle θ, defining position of a sterol molecule concerning specific regions of an antibiotic molecule: polyene region (yellow colour, angles 0–60° and 300–360°), in between (orange colour, angles 60–120° and 240–300°) and polyol region (red colour, angle 120–240°). Panel (**B**): a schematic depiction of the reaction coordinates (ξ, θ) used to define the relative position of the sterol and antibiotic molecules.

To thoroughly characterise interactions between antibiotic molecules and selected sterols in lipid bilayer environment, we have conducted extensive two-dimensional metadynamics (2D-MTD) computational studies for fourteen examined systems (CndD/Chol, CndD/Erg, iso-CndD/Chol, iso-CndD/Erg, ParA/Chol, ParA/Erg, iso-ParA/Chol, iso-ParA/Erg, ParB/Chol, ParB/Erg, iso-ParB/Chol, iso-ParB/Erg, AmB/Chol, AmB/Erg). As a result, free energy landscapes as a function of the centre-of-mass (COM) distance (ξ) between a given AHM/AmB and sterol, as well as the dihedral angle (θ) describing the relative orientation of AHM/AmB and sterol were determined. For more detailed visual explanation, please consult Figs. 2 and S1.

#### Candicidin D (CndD) and iso-candicidin D (iso-CndD)

**Figure 3:**
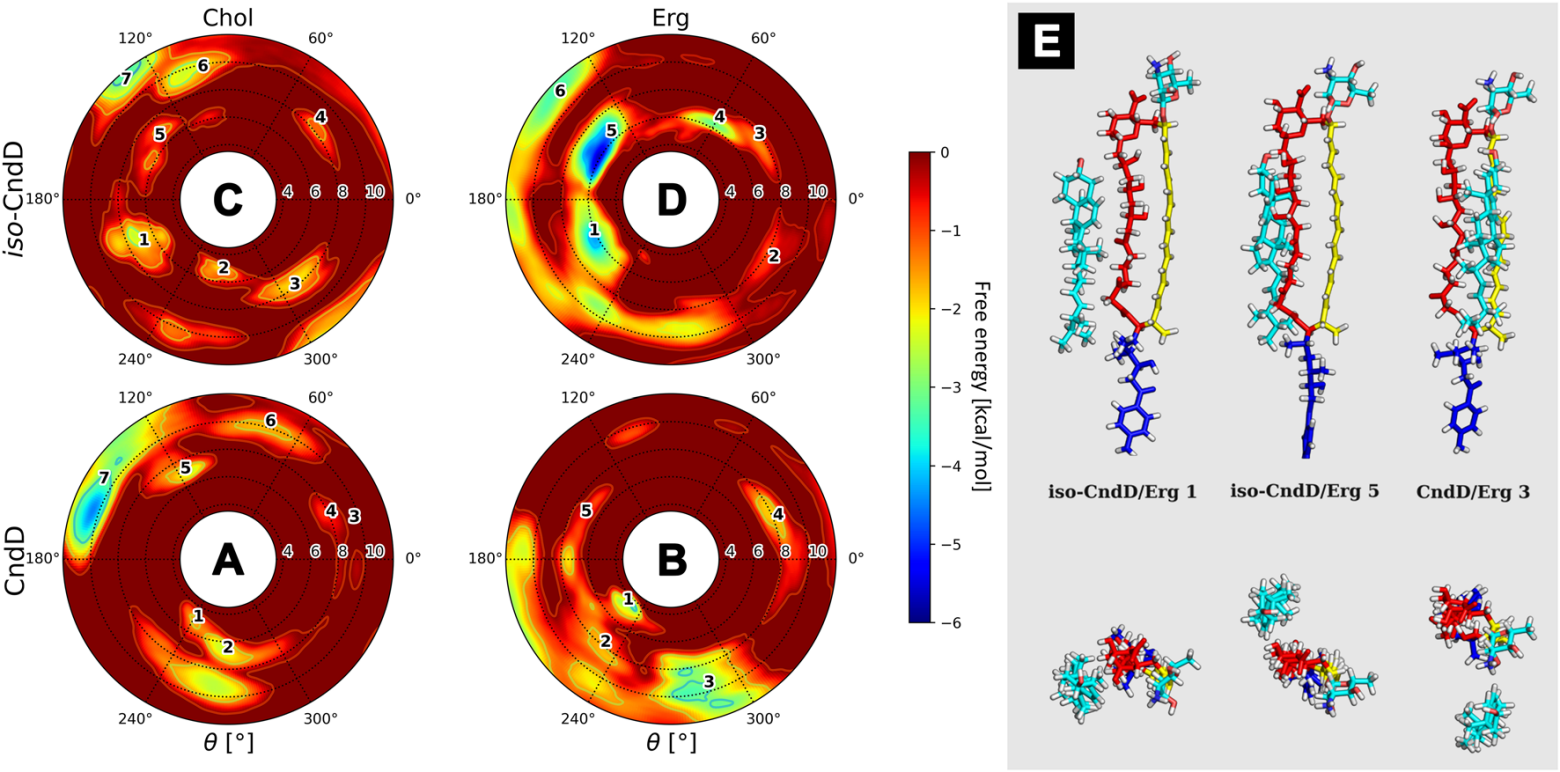
Free energy landscapes, calculated for candicidin D (panels **A** & **B**) and iso-candicidin D (panels **C** & **D**). Panel (**E**) contains representative structures of the (iso-)CndD/sterol complexes, extracted from selected energetic minima via cluster analysis. Number of a structure corresponds to the respective energetic minimum of a given system. Panel (**E**) colours: red—antibiotic’s polyol region; yellow—antibiotic’s heptaene chromophore; dark blue—alkyl-aromatic side chain; light blue—sterol molecule.

Free energy landscapes calculated for candicidin D and its all-trans photoisomer revealed the presence of deep energetic minima solely for iso-CndD/Erg system, while the rest of the antibiotic/sterol binary complexes did not exhibit strict conformational preferences. In case of iso-CndD/Erg ensemble, the ‘sweet spot’ for the ergosterol was revealed to be located near to the polyol fragment, at distance ξ ~ 6 Å (Fig. 3D,E, no. 1 & 5). Similar minima could be observed for iso-CndD/Chol system, yet they were considerably shallower in comparison to the iso-CndD/Erg complex (Fig. 3C, no. 1 & 5). Regarding CndD/Chol and iso-CndD/Chol systems, the deepest minima might be observed only around ξ ~ 10 Å (Fig. 3A,C, no. 7). At that distance, the complexes were already in a dissociated state, hence these shallow minima should not be considered relevant for the description of antibiotic/sterol interactions. Finally, the global energetic minimum in case of CndD/Erg system was located at macrolide ring plane, at the distance region ξ ~ 8–11 Å (Fig. 3B,E, no. 3). A similar minimum was present in case of CndD/Chol complex, yet less pronounced. Moreover, one could observe shallow minima of marginal significance at ξ ~ 8 Å regardless of the chromophore geometry and the sterol type, in θ spaces similar for all (iso-)CndD ensembles (Fig. 3, Fig. S2).

By comparing the four free energy landscapes, we can conclude that the isomerization of the heptaene chromophore—which results in its straightening—leads to the formation of more stable binary complexes with well-defined geometry, whereas the iso-CndD/Erg interactions seem to be slightly stronger than the ones between iso-CndD and Chol. Nevertheless, the differences between CndD/Chol and iso-CndD/Chol systems are in fact marginal, whereas iso-CndD/Erg complex exhibits noticeably different optimal geometry in comparison to the one presented by CndD/Erg duo.

#### Partricin A (ParA) and iso-partricin A (iso-ParA)

**Figure 4:**
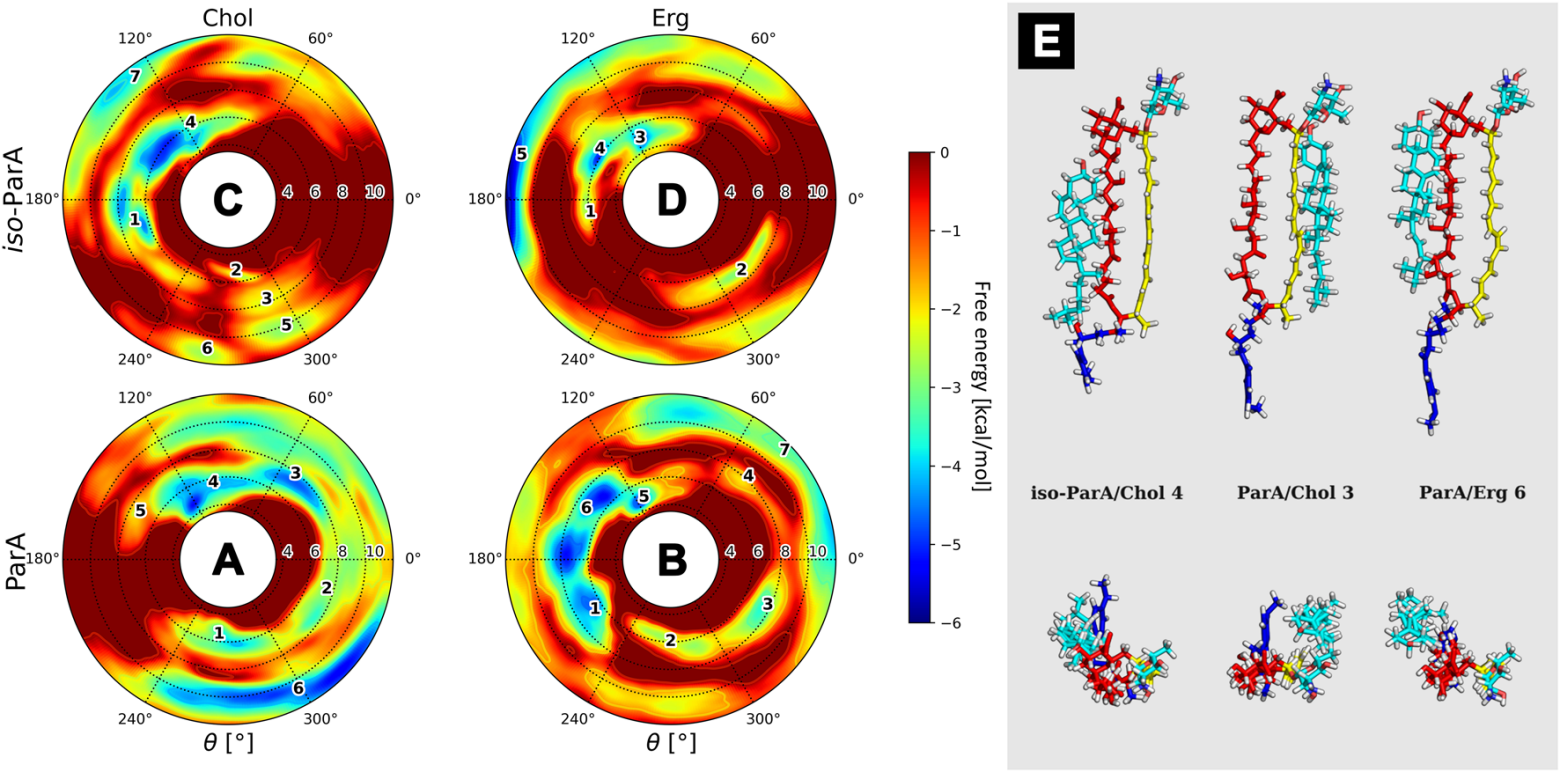
Free energy landscapes, calculated for partricin A (panels **A** & **B**) and iso-partricin A (panels **C** & **D**). Panel (**E**) contains representative structures of the (iso-)ParA/sterol complexes, extracted from selected energetic minima via cluster analysis. Number of a structure corresponds to the respective energetic minimum of a given system. Panel (**E**) colours: red—antibiotic’s polyol region; yellow—antibiotic’s heptaene chromophore; dark blue—alkyl-aromatic side chain; light blue—sterol molecule.

Free energy landscapes of native partricin A showed some remarkable differences, considering the type of a sterol embedded in the DPPC membrane. ParA/Chol interactions lacked specificity; the observed energy minima were wide and covered most of the θ space (Fig. 4A, no. 1–4,E), as cholesterol showed no preferential interactions with any particular region of the native ParA molecule, except for the strictly polyol region. On the contrary, ergosterol seemed to favour the very same region of the native partricin A that cholesterol avoided (Fig. 4B, no. 1,5 & 6,E); with addition of some more shallow and distant minima corresponding to the ones observed in case of ParA/Chol system (ξ ~ 8–11 Å). On the other hand, the landscapes obtained for iso-ParA/Chol and iso-ParA/Erg complexes were very similar, with both types of sterol choosing the proximity of polyol region at ξ ~ 6 Å (Fig. 4C, no. 1 & 4,D, no. 3 & 4,E) and occasionally interacting with macrolide ring planes at ξ ~ 8 Å. Notably, cholesterol and ergosterol seemed to utterly disfavour the polyene region of all-trans isomer of partricin A, since no energetic minima have been observed for θ ~ 0°.

Similarly to CndD and iso-CndD, photoisomerization process of ParA also impacts the way it interacts with sterol molecules in lipid bilayers. The most spectacular change may be observed for ParA/Chol and iso-Par/Chol systems (Fig. 4A,C), where the change of the geometry of the chromophore basically inverts the image of preferred θ distribution. Differences between ParA/Erg and iso-ParA/Erg complexes (Fig. 4B,D) are less pronounced, yet still visible. Once again, it might be stated that the more rigid all-trans geometry of an AHM species forces a more stable orientation of a sterol molecule with respect to the antibiotic (Figs. 4 and S3).

#### Partricin B (ParB) and iso-partricin B (iso-ParB)

**Figure 5:**
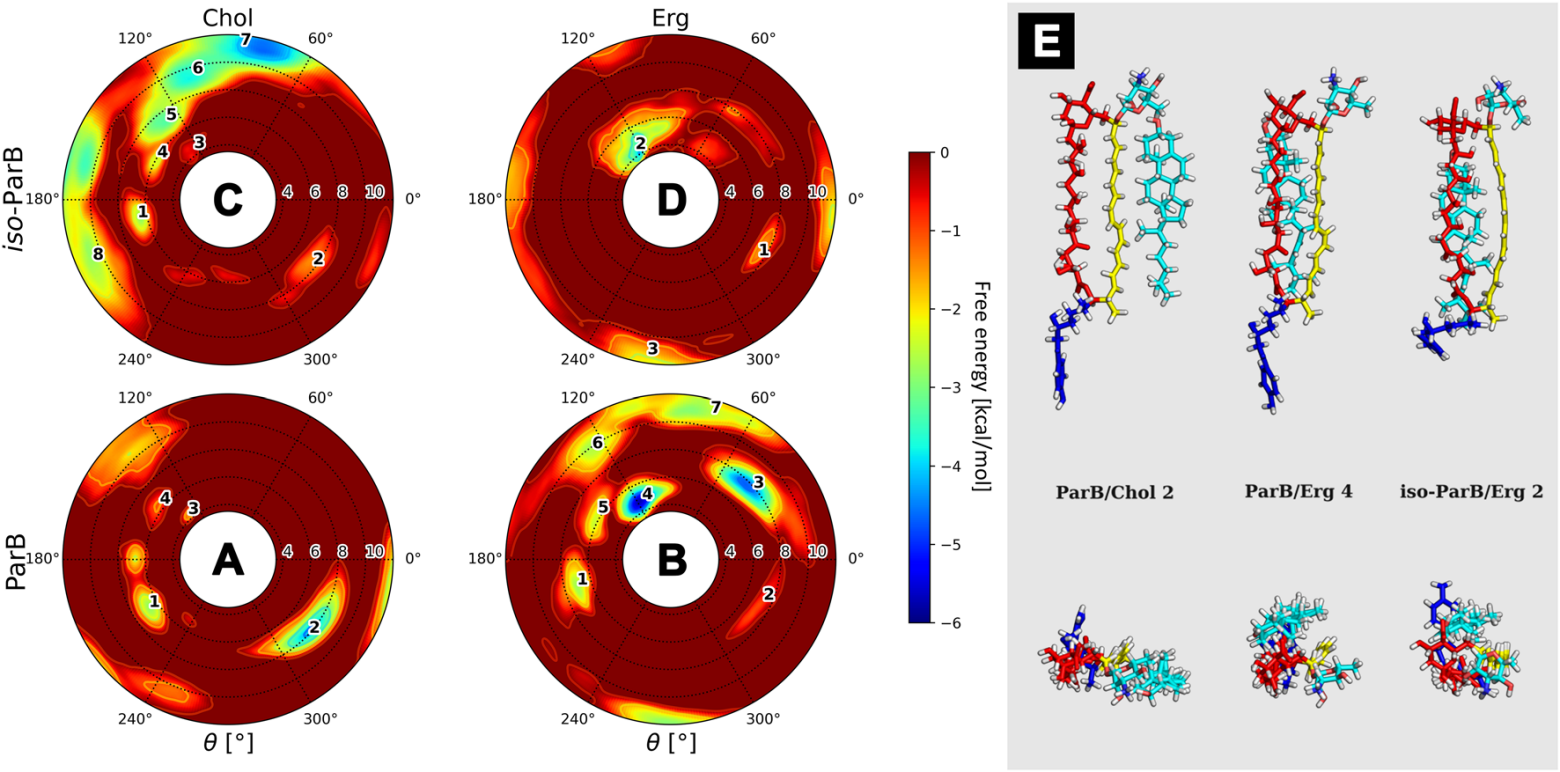
Free energy landscapes, calculated for partricin B (panels **A** & **B**) and iso-partricin B (panels **C** & **D**). Panel (**E**) contains representative structures of the (iso-)ParB/sterol complexes, extracted from selected energetic minima via cluster analysis. Number of a structure corresponds to the respective energetic minimum of a given system. Panel (**E**) colours: red—antibiotic’s polyol region; yellow—antibiotic’s heptaene chromophore; dark blue—alkyl-aromatic side chain; light blue—sterol molecule.

Absence of an N-terminal methyl moiety from the (iso-)partricin A skeleton, resulting in (iso-)partricin B molecule, had a significant impact on its interaction with sterols. Free energy landscapes obtained for the native partricin B revealed its almost specific (but weak) binding to cholesterol via polyene side of the macrolide ring plane (ξ ~ 8 Å, Fig. 5A, no. 2, E). In case of ergosterol, a similar minimum could be observed (Fig. 5B, no. 3), yet an additional, stronger and closer interaction was exposed near to the polyol part of ParB (ξ ~ 6 Å, Fig. 5B, no. 4,E). While the iso-ParB/Erg map was generally similar to its native counterpart (Fig. 5B,D), straightening of the polyene chromophore of ParB again caused the Chol molecule to favour an opposite region of the antibiotic (Fig. 5C), in comparison to the native ParB (Fig. 5A). Hence, the minima observed in case of iso-ParB/Chol (Fig. 5C) were quite shallow and similar to the ones displayed by iso-ParB/Erg duo (Fig. 5D,E).

Finally, comparison of ParB and iso-ParB revealed no significant differences in their interactions with ergosterol, despite generally weaker (~ 3 kcal/mol) binding of Erg to the all-trans partricin B isomer (compare Fig. 5B, no. 4,D, no. 2). At the same time, the ParB/Chol and iso-ParB/Chol systems behaved quite differently. As a result of a photoisomerization process, the change of the geometry of interactions between partricin B and cholesterol is not as spectacular as it was in case of partricin A, yet it is still well pronounced and generally similar (Fig. 5A,C). It is worth noting that iso-partricin B, similarly to its N-methylated iso-ParA cousin, is not particularly fond of sterol molecules trying to interact with its polyene region (Figs. 5 and S4).

#### The reference: amphotericin B (AmB)

**Figure 6:**
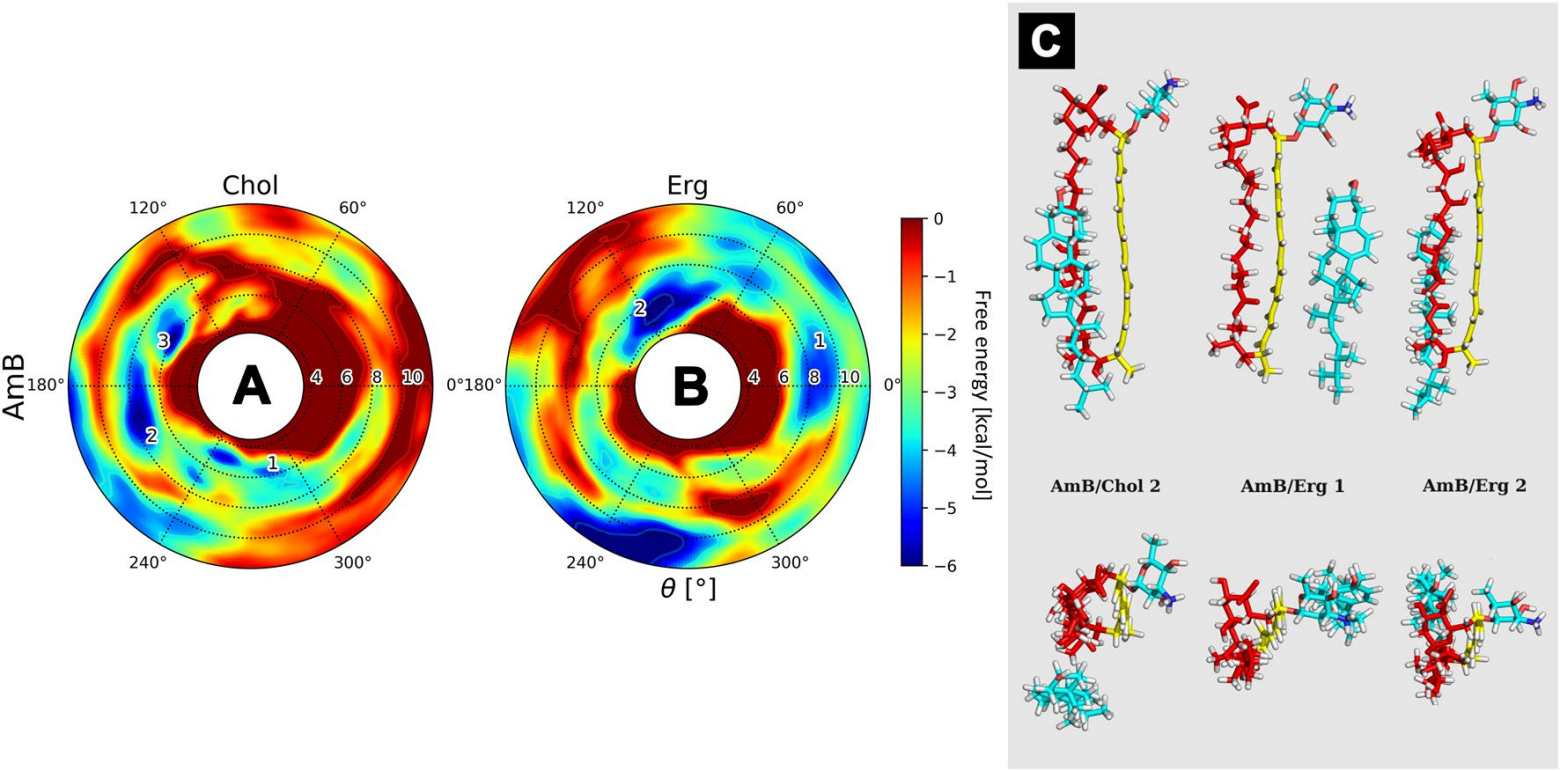
Free energy landscapes, calculated for amphotericin B (panels **A** & **B**). Panel (**C**) contains representative structures of the AmB/sterol complexes, extracted from selected energetic minima via cluster analysis. Number of a structure corresponds to the respective energetic minimum of a given system. Panel (**C**) colours: red—antibiotic’s polyol region; yellow – antibiotic’s heptaene chromophore; light blue—sterol molecule.

In comparison to AHM/sterol/DPPC systems, free energy landscapes obtained for amphotericin B (Fig. 6) also revealed the presence of several energetic minima in case of both AmB/Chol/DPPC and AmB/Erg/DPPC systems. In an AmB/sterol binary complex, the current paradigm arbitrarily locates both Chol and Erg molecules close to the polyene region of the antibiotic. Considering the results of our calculations, while for the AmB/Erg complex this mode of antibiotic/sterol binding still seemed to be among the ones preferred, the map obtained for the AmB/Chol binary complex (Fig. 6A) has strongly suggested that cholesterol molecule preferred the polyolic and macrolide ring plane regions of the antibiotic (θ ~ 120–300°) at ξ ~ 8–10 Å. Binding of cholesterol by the polyene side of AmB molecule was still a viable way of AmB/Chol interaction, yet the energetic minima at θ ~ 0° were considerably more shallow (~ 3 kcal/mol) in comparison to the ones aforementioned above. On the other hand, ergosterol (Fig. 6B) seemed to favour the polyene and macrolide ring plane regions of the amphotericin B at ξ ~ 6–8 Å, whereas the minimum at θ ~ 120° was slightly deeper (by ~ 1.5 kcal/mol) than the minimum at θ ~ 0°. The global energetic minimum for AmB/Erg ensemble was located at θ ~ 240° and ξ ~ 12 Å, therefore it corresponded to a dissociated state, hence it was not considered as relevant in this study.

The results obtained for AmB were coherent with the description of AHM/sterol interactions presented above. The preferred geometries of antibiotic/sterol interactions for both aromatic and non-aromatic heptaene macrolides were generally similar, yet the convergence of the 2D-metadynamics calculations was achieved much later in case of AHMs. In order to search for a possible source of this contrast, we have focused on a major structural difference between AHMs and AmB: the alkyl-aromatic side chain, attached to the macrolide ring system of the former.

### The alkyl-aromatic side chain of aromatic heptaene macrolides

The length of the greater axis of a macrolactone ring of aromatic heptaene macrolides (AHMs) is, more or less, equal to the thickness of a single leaflet of a DPPC bilayer, containing 30 mol % of cholesterol or ergosterol. While DPPC/Chol and DPPC/Erg membranes display slightly different properties, such as fluidity and total thickness^24^, in both cases the alkyl-aromatic side chain of AHMs has a potential of a penetration of an opposite leaflet of a bilayer. Such an interaction with a second leaflet could have an enormous impact on antibiotic’s interaction with sterols and its general dynamics in a membrane. To examine this impact, we introduced an additional geometric parameter, further referred to as δ, being the distance between the centre of mass (COM) of aromatic carbon atoms of the side chain and the COM of phosphorus atoms of a leaflet opposite to the layer in which the antibiotic was embedded. While for δ > 26 Å, the side chain would not interact with a second layer; whereas for δ < 22 Å the side chain was fully embedded in an opposite leaflet of a bilayer. For 22 Å < δ < 26 Å, the alkyl-aromatic moiety of AHMs was located between the layers of a membrane. Percentage values of the simulation time while the side chain was present in a second leaflet of a given system and the antibiotic/sterol complex was dissociated (θ = 0–360°; ξ ≥ 10 Å) were listed in Table 1.

**Table 1:** Percentage values of the simulation time while the side chain was embedded in an opposite leaflet of a given system (calculated for the dissociated antibiotic/sterol complexes).

	*Isomeric (all-trans) form*		*Native (cis-trans) form*	
	Chol	Erg	Chol	Erg
CndD	59.66%	69.71%	49.31%	67.18%
ParA	16.28%	58.29%	46.82%	73.58%
ParB	25.81%	22.35%	44.72%	47.82%

#### Candicidin D and iso-candicidin D

**Figure 7:**
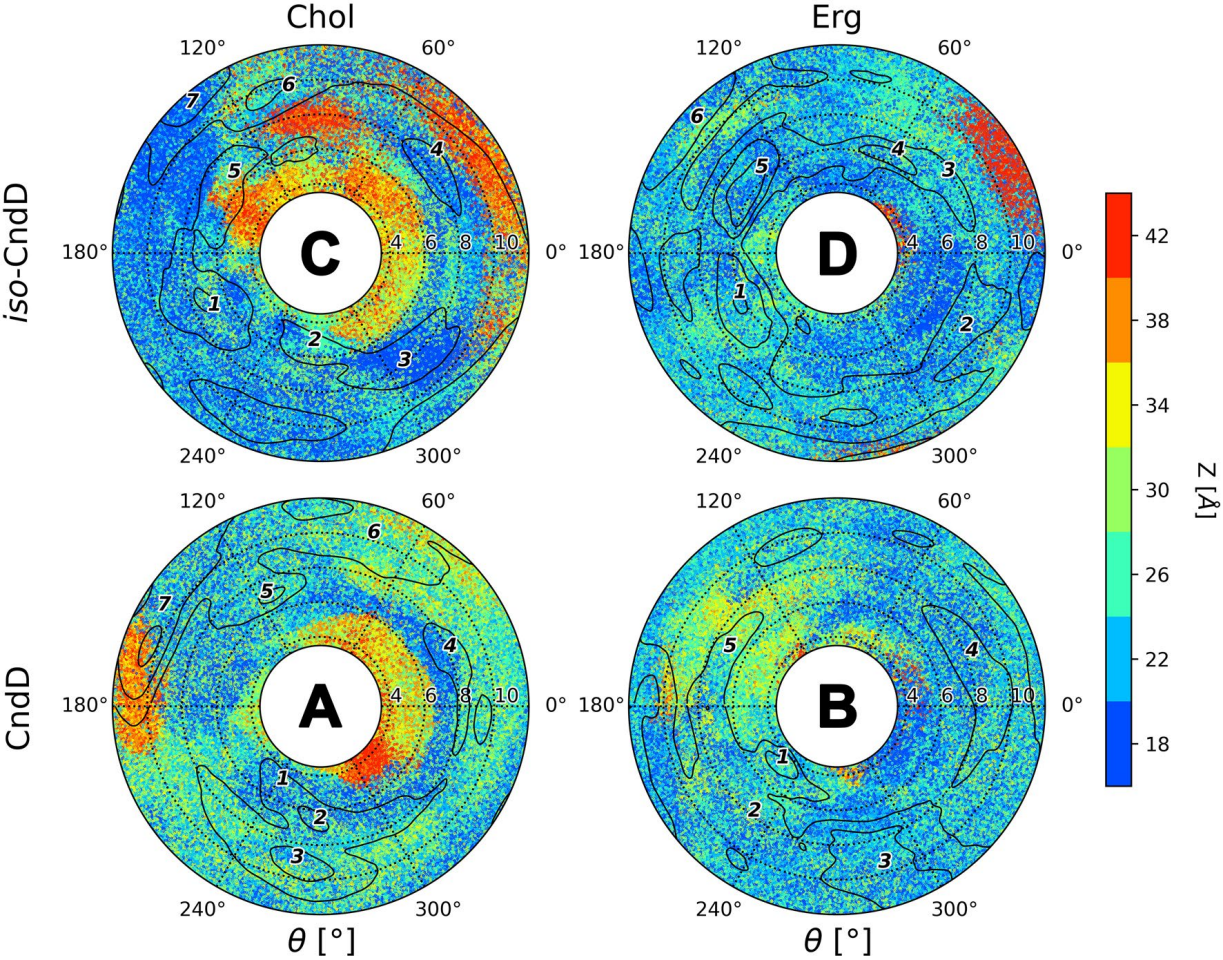
Interactions of candicidin’s D (**A** & **B**) and iso-candicidin’s D (**C** & **D**) side chain with an opposite leaflet of a lipid bilayer.

Comparison of δ landscapes for CndD and iso-CndD (Fig. 7) revealed minor differences between native and isomeric forms in both Chol and Erg membranes. Hence, one could conclude that straightening of the heptaene chromophore of candicidin D did not have a substantial impact on sidechain’s interaction with a second layer of a given membrane. However, it has been observed that in the DPPC/Erg membranes (Fig. 7B,D) the alkyl-aromatic moiety of CndD and iso-CndD did interact a bit more often with a second leaflet in comparison to their DPPC/Chol counterparts (Fig. 7A,C). Moreover, considering the free energy landscapes presented above (Fig. 3), it could be stated that the most important energetic minima (also outlined at Fig. 7) appeared in the ξ and θ spaces in which the side chain of both versions of candicidin D was interacting with a second layer of a membrane or, at least, was located between the leaflets.

#### Partricin A and iso-partricin A

**Figure 8:**
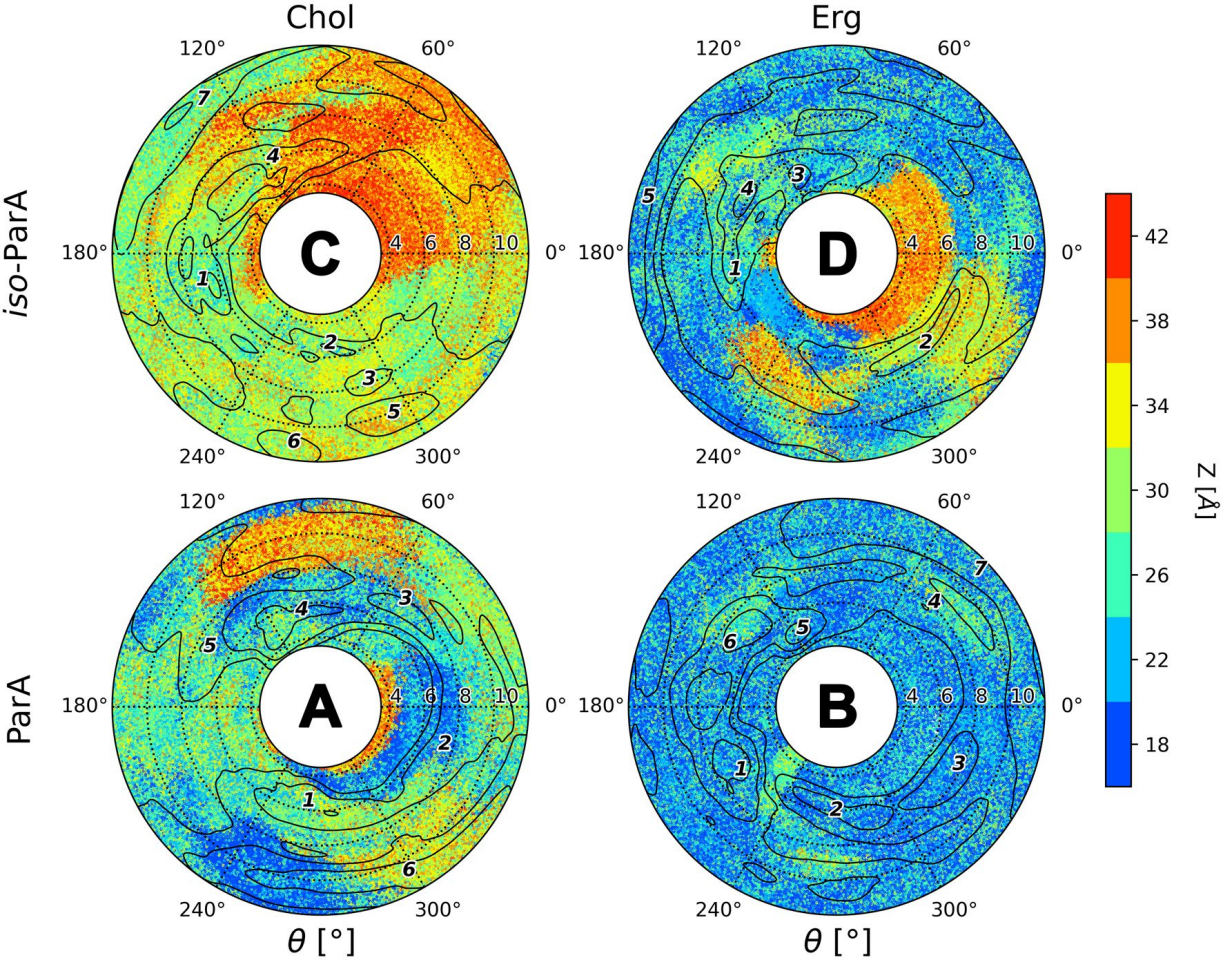
Interactions of partricin’s A (**A** & **B**) and iso-partricin’s A (**C** & **D**) side chain with an opposite leaflet of a lipid bilayer.

Δ distributions computed for ParA and iso-ParA displayed the most spectacular differences observed for a single antibiotic in this study. While for the native partricin A (Fig. 8A,B) the results were, more or less, in line with native candicidin D (Fig. 7A,B), the all-trans partricin A has painted a substantially different image. The alkyl-aromatic moiety of iso-ParA has found the opposite leaflet of the DPPC/Chol membrane particularly repelling, spending merely a fraction (~ 16%) of the simulation time in its second layer (Table 1, Fig. 8C). Meanwhile, the sidechain of the very same molecule was a frequent guest in the second leaflet of the ergosterol membrane (Table 1, Fig. 8D), although the time of this interaction (~ 58%) was significantly shorter in comparison to its native counterpart (~ 74%). Finally, a trend similar to the one displayed by CndD and iso-CndD was observed—the presence of the energetic minima (Fig. 4) was correlated with the interaction of the antibiotic’s side chain with a second layer of a membrane (or its location between the leaflets) in all four simulation (iso-)ParA systems.

#### Partricin B and iso-partricin B

**Figure 9:**
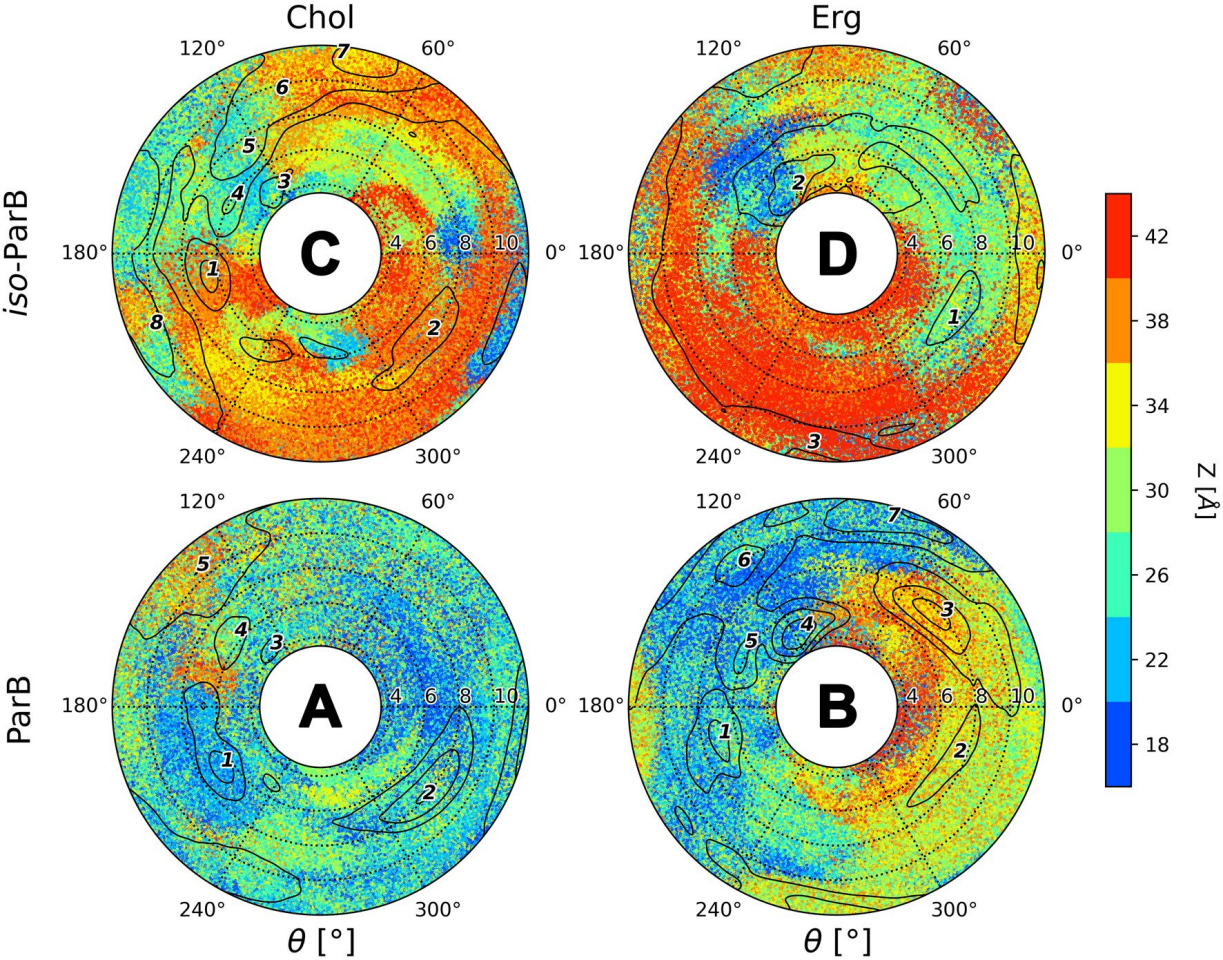
Interactions of partricin’s B (**A** & **B**) and iso-partricin’s B (**C** & **D**) side chain with an opposite leaflet of a lipid bilayer.

The alkyl-aromatic side chain of the native version of partricin B has spent around half of the simulation time in the second leaflet of both cholesterol and ergosterol membranes (Table 1, Fig. 9A,B). This result was comparable to the one obtained for native CndD, yet the δ distribution was a bit different—in case of ParB the side chain was more often located in the same layer as the rest of the antibiotic molecule, especially in the DPPC/Erg system (Fig. 9B). In contrast, results obtained for iso-ParB embedded in Chol and Erg membranes (Fig. 9C,D) were comparable to the DPPC/Chol/iso-ParA setup (Fig. 8C), yet in case of iso-ParB the alkyl-aromatic moiety has presumably enjoyed the close company of the rest of the molecule even more, for the most of the time locating itself in the same leaflet (Table 1). Interestingly, considering free energy landscapes for (iso-)ParB (Fig. 5), once again no important energetic minima were observed in the ξ and θ spaces where the side chain was located in an ‘upper’ leaflet of any studied system.

### Orientation on an alkyl-aromatic side chain of aromatic heptaene macrolides in relation to a macrolactone ring

#### Possible conformations of the side chain

**Figure 10:**
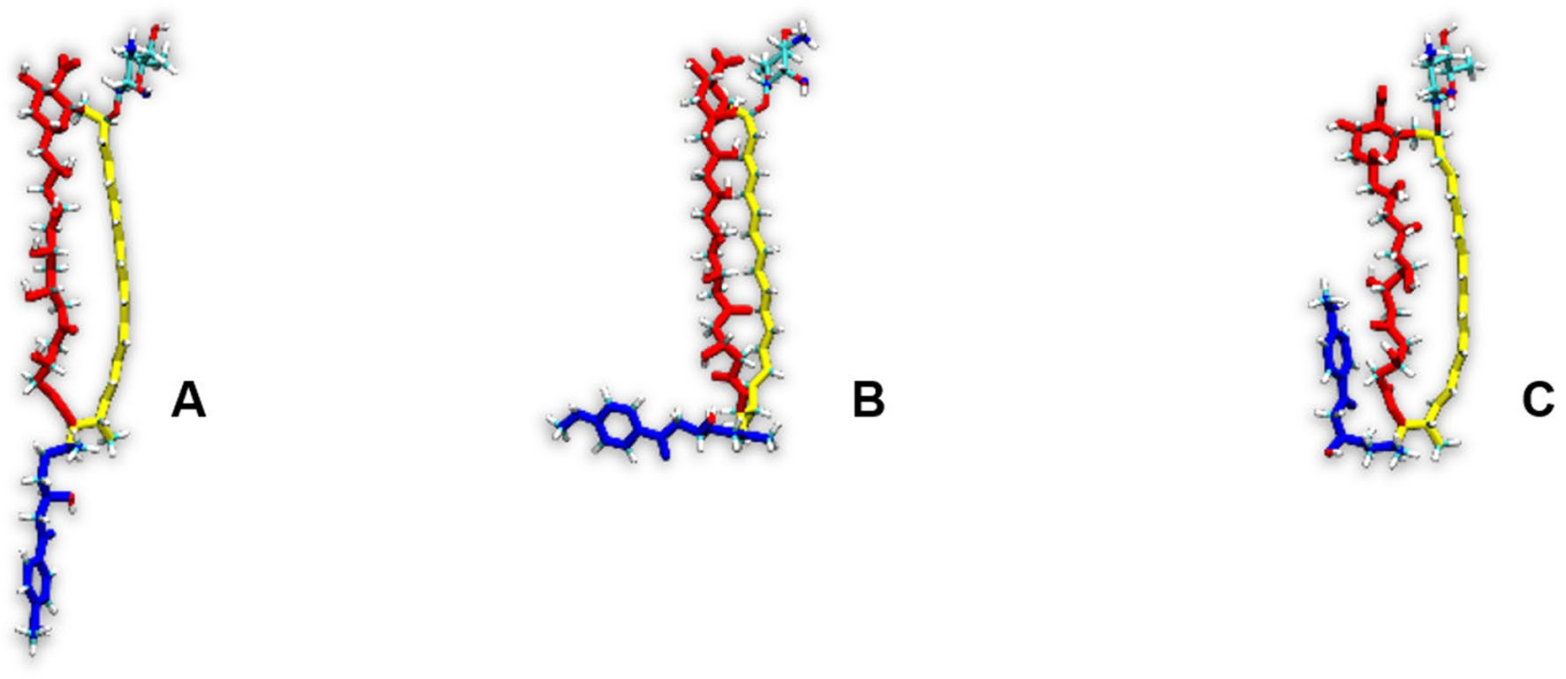
Orientation of an AHM’s alkyl-aromatic sidechain in relation to the rest of the molecule: (**A**)—aligned; (**B**)—bent; (**C**)—tucked up.

One could reasonably state that the presence of the side chain of a given AHM in an ‘upper’ leaflet of a membrane might not necessarily mean that the rest of the molecule was also embedded herein. For instance, in such a case the macrolactone ring could be lifted up and therefore ‘stick out’ from the membrane, while the whole molecule could in fact remain stretched. In order to deal with that possibility, trajectories resulting from 2D-MTD simulations were also analysed with respect to the orientation of an alkyl-aromatic side chain of AHMs in relation to a macrolide ring. For each considered antibiotic molecule, the values of the angle ϕ, defined—in each case—by the nitrogen atom of the alkyl-aromatic moiety, C36 and C22 (Fig. 1), were traced over time. The analyses revealed that the three major conformations of a side chain could be observed: antiparallel to the major axis of a macrolide ring, a.k.a. aligned (ϕ ~ 180°, Fig. 10A); perpendicular to the major axis of a macrolide ring, a.k.a. bent (ϕ ~ 90°, Fig. 10B) and parallel to the major axis of a macrolide ring, a.k.a. tucked up (ϕ ~ 0°, Fig. 10C).

#### Candicidin D and iso-candicidin D

**Figure 11:**
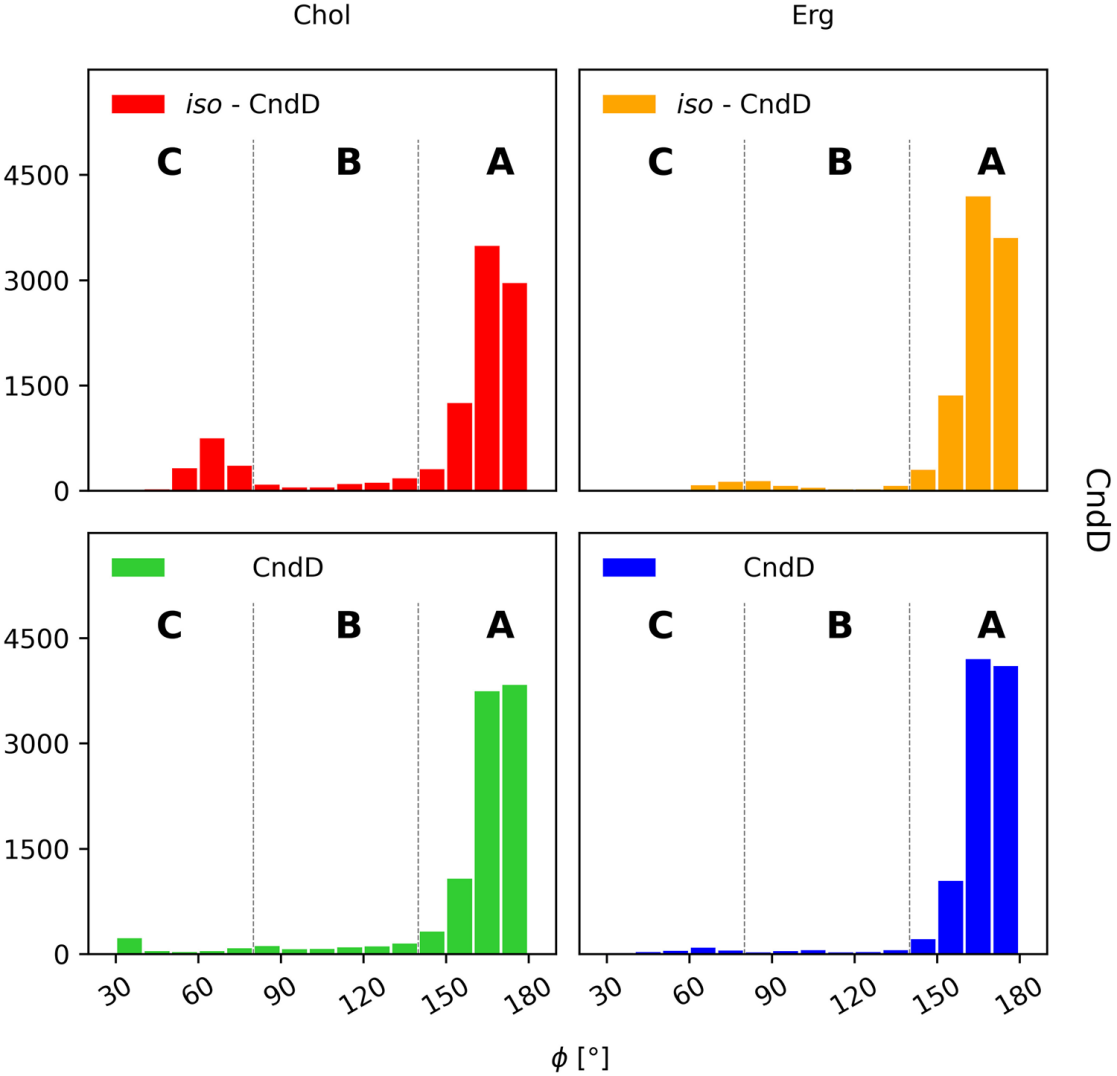
Distribution of the orientation of the (iso-)candicidin’ D alkyl-aromatic sidechain in relation to the rest of the molecule: (**A**)—aligned; (**B**)—bent; (**C**)—tucked up.

In case of CndD and iso-CndD, the alkyl-aromatic side chain assumed one dominant, aligned conformation (ϕ ~ 180°, Fig. 10A) in all four simulation systems (Fig. 11). The share of the other conformations was marginal in all cases. Hence, straightening of the heptaene chromophore seemed to have no impact on the alkyl-aromatic moiety’s dynamics and orientation.

#### Partricin A and iso-partricin A

**Figure 12:**
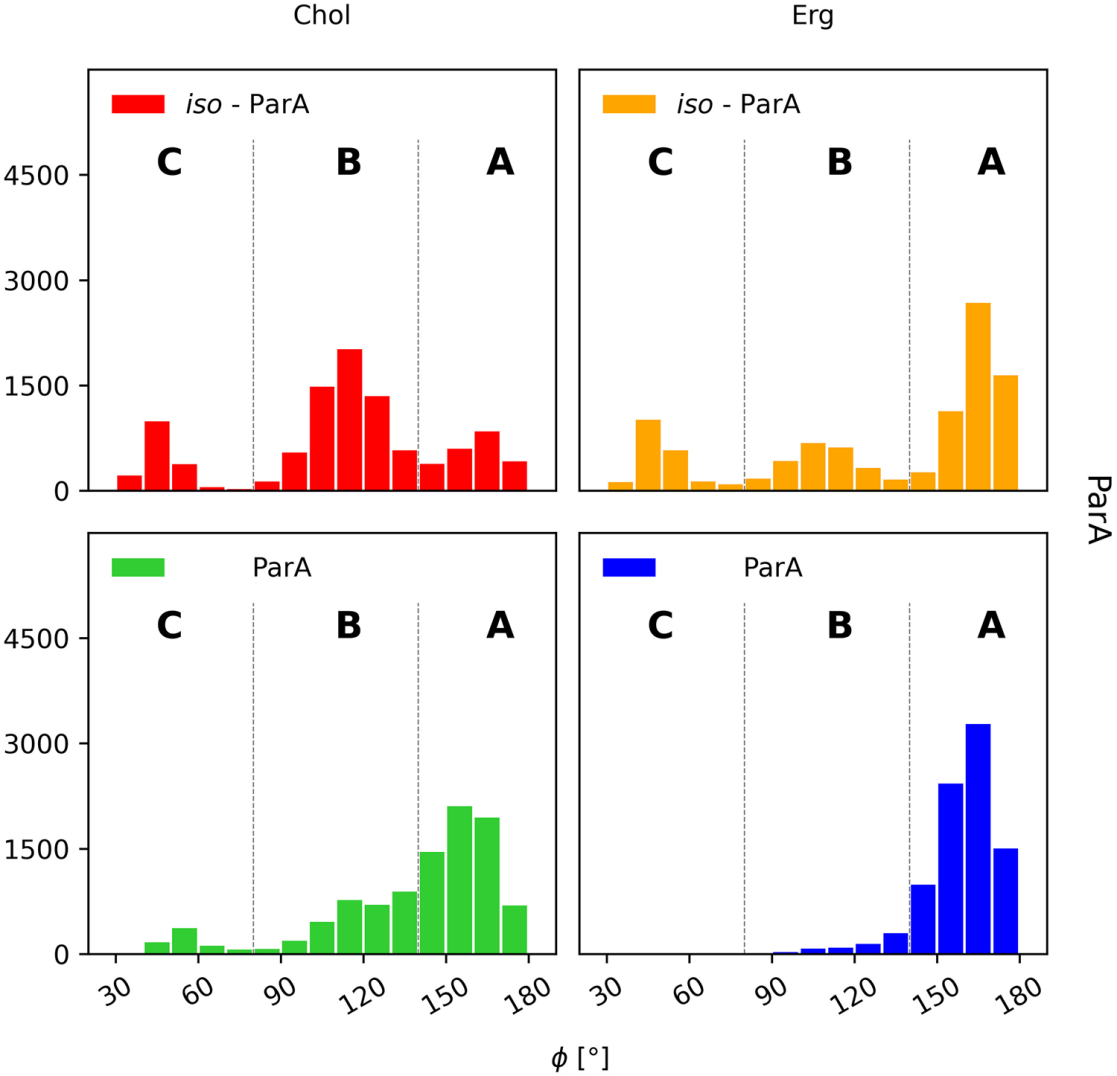
Distribution of the orientation of the (iso-)partricin’s A alkyl-aromatic sidechain in relation to the rest of the molecule: (**A**)—aligned; (**B**)—bent; (**C**)—tucked up.

Analyses performed for the native ParA revealed that the distribution of the orientation of alkyl-aromatic moiety was slightly more diversified in comparison to CndD. Nevertheless, the aligned conformation (ϕ ~ 180°, Fig. 10A) was still dominant (Fig. 12). On the contrary, iso-ParA presented a fundamentally different dynamics of its alkyl-aromatic side chain. While interacting with cholesterol, the bent conformation (ϕ ~ 90°, Fig. 10B) was the one most commonly observed. In ergosterol membrane, while the side chain of iso-ParA was aligned (ϕ ~ 180°, Fig. 10A) in most cases, the bent (ϕ ~ 90°, Fig. 10B) and tucked up (ϕ ~ 0°, Fig. 10C) conformations have also appeared quite frequently. In the end, the differences between ParA and iso-ParA were quite pronounced, which pointed to a substantial impact of the geometry of the chromophore on the side chain’s behaviour.

#### Partricin B and iso-partricin B

**Figure 13:**
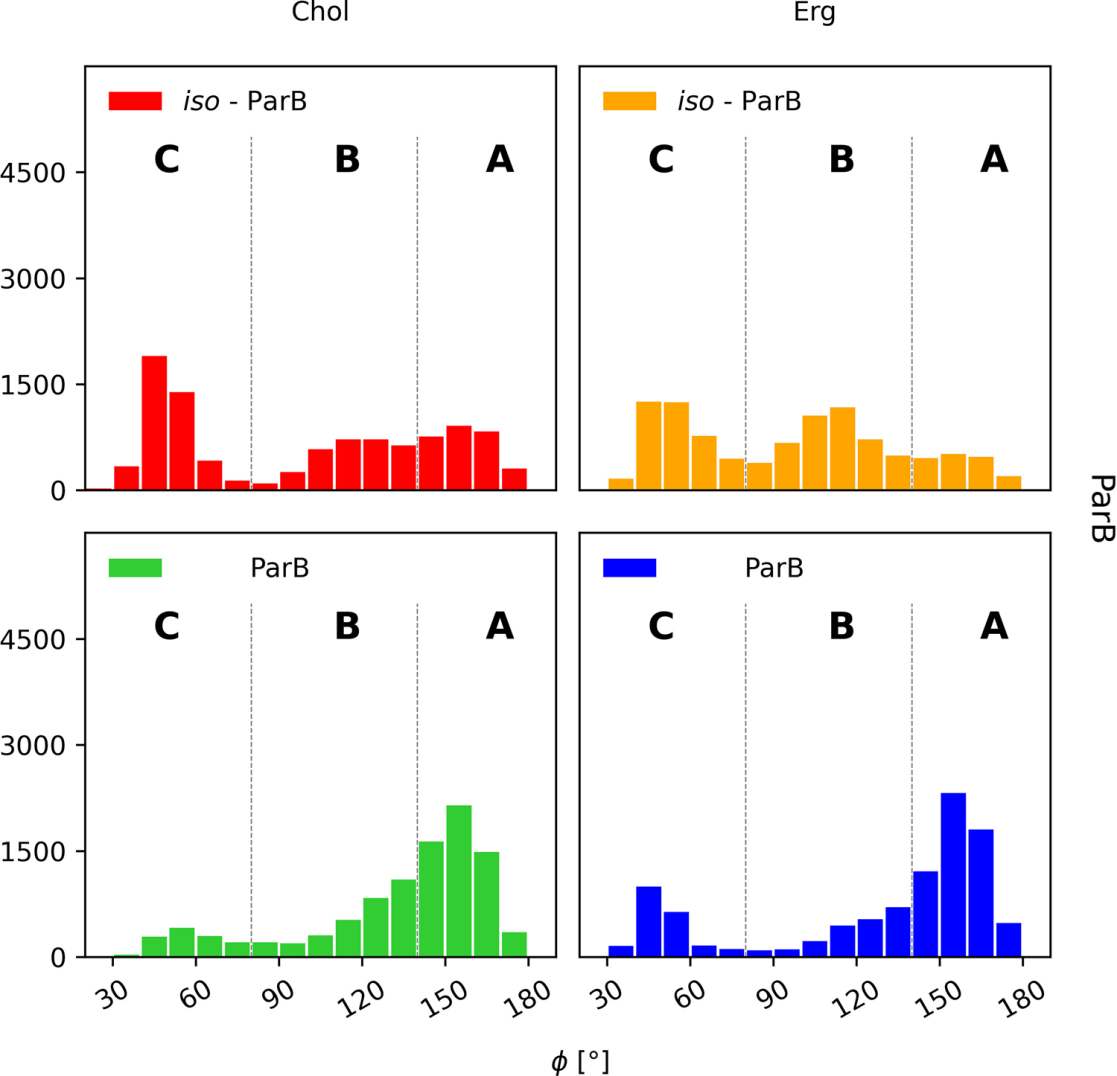
Distribution of the orientation of the (iso-)partricin’s B alkyl-aromatic sidechain in relation to the rest of the molecule: (**A**)—aligned; (**B**)—bent; (**C**)—tucked up.

Native partricin B presented a distribution of the orientation of its alkyl-aromatic moiety (Fig. 13) similar to the one of native partricin A, except for indisputably more frequently observed tucked up (ϕ ~ 0°, Fig. 10C) conformation. All-trans partricin B showed a behaviour like no other molecule discussed so far—tucked up (ϕ ~ 0°, Fig. 10C) and bent (ϕ ~ 90°, Fig. 10B) conformations were the most common in both cholesterol and ergosterol membranes. In case or iso-ParB/Erg interactions, no dominant conformation of the antibiotic’s side chain could be pointed at—the distribution was flattened and displayed no global maximum. Hence, one could reasonably conclude that the impact of the partricin’s B chromophore geometry on the side chain’s dynamics in Chol/Erg lipid membranes was the most spectacular one of all cases presented herein.

## Discussion

Taking into account the free energy landscapes obtained for twelve AHM simulation systems (Figs. 3, 4 and 5), we have concluded that the least contrasting—in terms of energetic description and the spatial definition of the resulting complexes—were the antibiotic/sterol interactions displayed by candicidin D (Fig. 3), while the straightening of its heptaene chromophore did not result in any crucial changes in that regard. There were no fundamental differences in CndD/Chol and CndD/Erg interactions, whereas the iso-counterparts of these systems yielded similar results with slightly deeper energetic minima. A different story could be told of partricin B (Fig. 5), since its all-trans isomer preferred a cholesterol molecule approaching from an opposite side of the antibiotic in comparison to the native ParB. The ParB/Erg and iso-ParB/Erg systems behaved quite similarly, although the latter interaction was weaker. Finally, partricin A (Fig. 4) turned out to be the most remarkable in terms antibiotic-sterol interactions, since it has presented more, vast and deeper energetic minima, whereas the change of the geometry of ParA’s chromophore practically inverted the geometry of its interactions with cholesterol (compare Fig. 4A,C). Although the observed energetic minima for antibiotics’ isoforms and their comparison with minima observed for their native counterparts were far from enough to solely explain the reported gains in vitro selective toxicity indices (STIs) for all-trans aromatic heptaene macrolides^22^, the biggest change in both free energy landscapes and STIs were in fact displayed for ParA/iso-ParA pair. Having in mind that the least pronounced changes in the free energy landscapes and STIs were observed for native and iso-candicidin D, the results presented herein strongly support the claim that the energy and geometry of the antibiotic/sterol complexes indeed have an impact on AHMs’ biological activity. The in vitro selective toxicity indices for (iso-)AMHs and AmB were reported before by our team^22^, yet the key biological data from that study was displayed herein as Fig. S15.

In the end, it might be concluded that the better spatial and energetical definition of iso-AHM/sterol complexes, resulting from the straightening of the AHMs’ chromophores, do in fact contribute to the in vitro selective toxicity indices of these antibiotics, yet it is unreasonable to derive all aspects of these drugs’ biological activity from a behaviour of a single molecule in a lipid bilayer environment.

**Figure 14:**
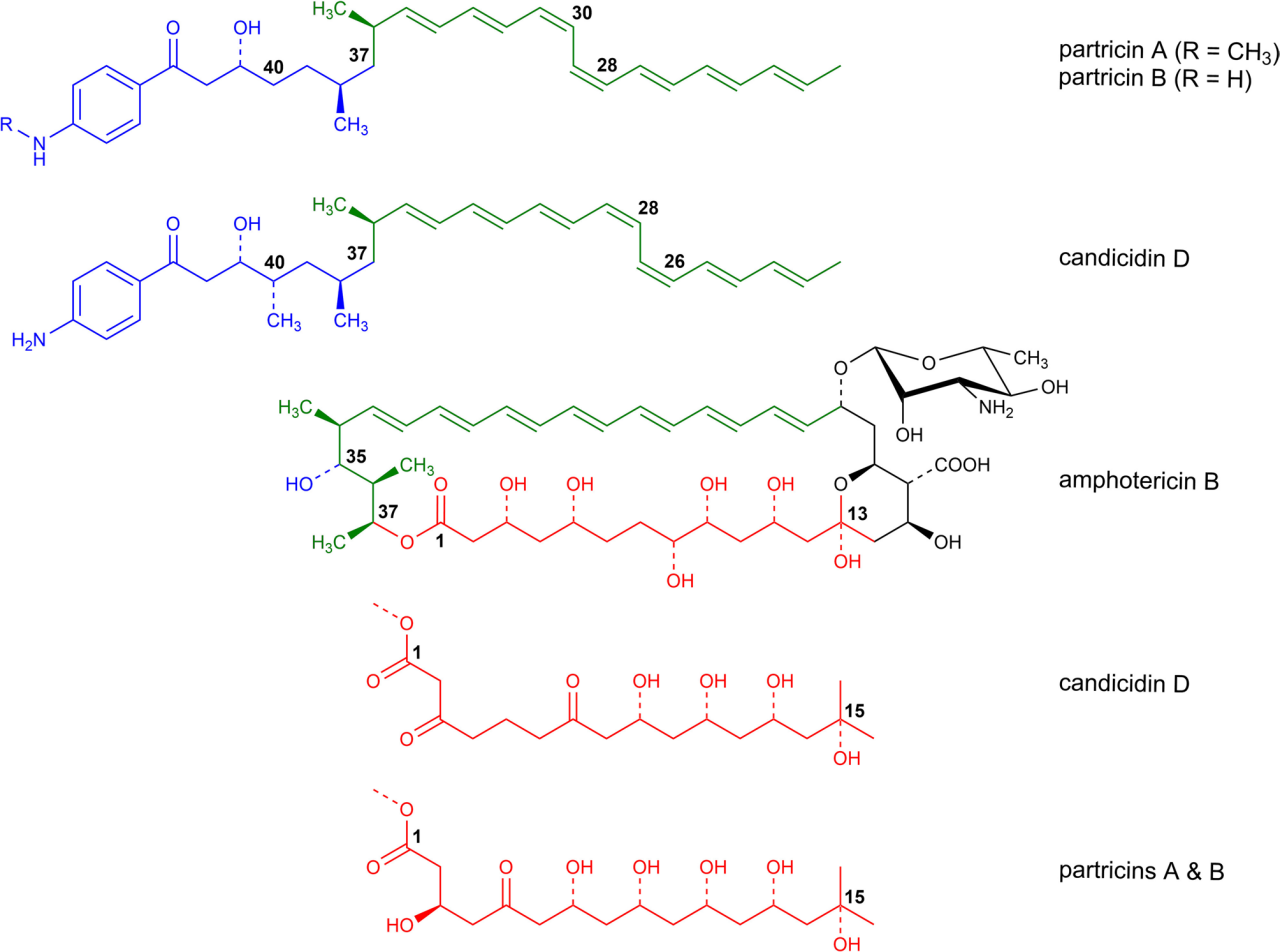
Structural differences between the native AHMs and AmB. Colours: red—polyol region; green—chromophore; blue—alkyl-aromatic sidechain.

One might speculate why the observed differences between (iso-)candicidin D and the (iso-)partricins were so evident. As it goes for the antibiotic/sterol interactions, highlighted by the free energy landscapes, one of the main reasons seemed to be the constitution of the polyol regions of the antibiotics (Fig. 14). Candicidin D contains far less hydroxyl functions in the C1-C15 fragment in comparison to the both partricins, hence its hydrogen-bond network is significantly less extensive. As a consequence, the macrolactone ring of candicidin D is notably less rigid than the one of both partricins and the alteration of the candicidin’s chromophore is not much of an improvement in this area. Therefore, no substantial differences between CndD and iso-CndD were identified in any of the performed analyses. Coherently, CndD/iso-CndD pair did not show any significant differences in the in vitro assessments of their selective toxicity.

The surprising conclusion from these studies is that the sterols preferably locate themselves near the polyol region of the (iso-)AHM molecules and/or near the planes between polyol and polyene regions (Figs. 3, 4 and 5). This finding is in disagreement with a ‘canonical’, relative orientation of AmB/sterol complex, where a sterol molecule is located near the polyene part of the antibiotic^23^. In order to settle this argument, we have also examined the AmB/Chol/DPPC and AmB/Erg/DPPC ensembles (Fig. 6). The surprising results was that, even in case of AmB, the antibiotic’s most preferred region of sterol binding was the macrolide ring plane, whereas cholesterol would also consider the AmB’s polyolic region, while ergosterol would also exhibit a relatively strong preference to the heptaene chromophore. These results contradict the current paradigm of AmB’s polyene chromophore being—by far—the default binding site of both Chol and Erg. The reality seems to be much more complicated and the Chol's & Erg’s preferred sites of binding to AmB might play a role in the molecular mode of action of this drug.

The alkyl-aromatic moiety of aromatic polyene macrolides displayed three possible orientations in relation to the antibiotics’ macrolide ring: aligned, bent and tucked up (Figs. 10, 11, 12 and 13). Careful examination of the side chain’s behaviour in relation to the membranes themselves (Figs. 7, 8 and 9) revealed that the aforementioned three conformations were in agreement with the location of the alkyl-aromatic moiety: in the lower leaflet of a lipid bilayer, in between and in the upper layer (i.e. the same layer in which the rest of molecule resided), respectively. While comparing Figs. 7, 8, 9, 11, 12 and 13, it is obvious that the side chains of candicidin D, partricin A and partricin B behave quite differently and the source of their distinct dynamics must be directly related to their slightly different structures (Fig. 14). The structural, discriminating factors of the side chains are the two methyl groups: one residing on aniline-type nitrogen of solely partricin A and second, methyl moiety exclusively bound to C40 of candicidin D. Hence, in order to reveal the role of each methyl group, we can compare: 1) CndD and ParB, considering the presence/lack of C40 methyl substituent, and 2) ParA and ParB, highlighting the function of N-terminal methyl on the side chain. Comparison of ϕ distributions calculated for CndD and ParB revealed a much more marked mobility of partricin’s B alkyl-aromatic moiety. This effect was even more explicit when iso-CndD and iso-ParB systems were considered (Figs. 7C,D and 9C,D). It might be concluded that C40-bound methyl introduces steric hindrance to the side chain, constraining its conformational freedom and forcing its aligned orientation (Fig. 11). On the other hand, while studying the δ landscapes prepared for ParA and ParB (Figs. 8 and 9, respectively), it was clearly visible that the presence of N-terminal methyl on ParA’s side chain promoted its interactions with the opposite leaflets of the membranes. The biggest difference could be observed while confronting the DPPC/Erg/iso-ParA and DPPC/Erg/iso-ParB ensembles (compare Figs. 8D and 9D). As listed in Table 1, it was a ~ 58% versus ~ 22% disparity in the simulation time spent by a side chain in a lower layer of a membrane, respectively. It could be at least partially explained by the fact that a methyl moiety bound to aniline-type nitrogen not only raises the total lipophilicity of the side chain, but also changes the electronic properties of the aromatic moiety. In the end, while comparing the results obtained for all 12 studied AHM systems, one could state that in order to observe a reasonable differences in native/isomeric and cholesterol/ergosterol ensembles, the alkyl-aromatic moiety should maintain its mobility, yet also preserve its relatively high lipophilicity. Therefore, the presence of C40 methyl seems disfavourable, while the N-terminal methyl moiety makes the side chain much more universal in its interactions with lipid bilayers. Hence, the structure of the side chain of (iso-)partricin A was dubbed to be an optimal among the ones examined in this study.

The general role of an alkyl-aromatic moiety of aromatic polyene macrolides still remains not fully understood. A correlation between its aligned conformation and energetic minima observed in free energy landscapes hints that the side chain might serve as a molecular anchor, stabilizing its position in a membrane, aiding the AHM/sterol complex formation, and—in extent—the assembly of a potential transmembrane channel. Possibly, it could also promote the initial step of embedding an antibiotic molecule into a lipid bilayer. The detailed computational and experimental studies on AHM antibiotics entering various versions of membranes, their orientation herein and assembly into higher order structures are on the way.

## Methods

### Molecular models

The basic forcefield parameters for the atomistic models of the native (cis-trans) candicidin D (CndD), partricin A (ParA), partricin B (ParB) and their isomeric (all-trans) counterparts were taken from CHARMM Generalized ForceField (CGenFF)^25^ using the web interface, available at paramchem.org. Hence, the partial atomic charges for all six molecules were recalculated ab initio at the MP2/6-31G* level of theory. In the next step, dihedral angles defined by the heavy atoms of the C35-C4 fragment of all molecules were re-parameterized by fitting to energy profiles obtained from quantum calculations, since they scored high penalties during the application of CGenFF. This was carried out using GAUSSIAN09 implementation of a dihedral scanner at the MP2/6-31G* level of theory^26^. The dihedral step was equal to 5 degrees. The resulting energetic profiles were used to fit the dihedral parameters with ForceField ToolKit (ffTK)^27^ hosted by VMD software^28^. Additionally, parameters defining the dihedrals constituting the glycosidic bond between the aglycone and the mycosamine moiety were taken from previous studies on amphotericin B^29^, as these fragments of CndD, ParA, ParB and their all-trans isoforms are structurally identical to the one of AmB.

The forcefield parameters for (iso-)CndD, (iso-)ParA and (iso-)ParB, including refined partial atomic charges and dihedrals, were attached as Supplementary Data in a form of GROMACS topology files.

The refined CHARMM forcefield parameters for amphotericin B (AmB) were taken from previous studies^17^.

### Preparation of twelve simulation systems

Using the CHARMM Membrane Builder^30^, single molecules of the native and isomer aromatic heptaenes (AHMs), as well as amphotericin B (AmB) were separately embedded in a dipalmitoylphosphatidylcholine lipid bilayer (DPPC) containing 30 mol % addition of cholesterol (Chol) or ergosterol (Erg) molecules respectively, resulting in 14 simulating systems in total (candicidin D with cholesterol, candicidin D with ergosterol, iso-candicidin D with cholesterol, iso-candicidin D with ergosterol, partricin A with cholesterol, partricin A with ergosterol, iso-partricin A with cholesterol, iso-partricin A with ergosterol, partricin B with cholesterol, partricin B with ergosterol, iso-partricin B with cholesterol, iso-partricin B with ergosterol, amphotericin B with cholesterol, amphotericin B with ergosterol). Then, the generated systems were solvated with water (~ 4400 water molecules in average) and ions corresponding to concentration of 0.15 mol/dm^3^ to provide physiological ionic strength. The CHARMM36 force field^31^ was used for AHMs, AmB, sterols, DPPC and ions and the TIP3P model^32^ was used for water.

### Molecular dynamics simulations

All simulations were implemented in GROMACS version 2020.4^33^. The simulation temperature was kept at 310 K by the Nosé-Hoover thermostat^34^, the pressure was maintained at 1 bar with the Parrinello-Rahman method^35^. PBC were employed for all simulations. The particle mesh Ewald (PME) method was used for long-range electrostatic interactions with 12 Å cut-off and 1.2 Å FFT grid spacing^36^, while the Lennard-Jones interactions were smoothly switched off between 10 and 12 Å. After the conventional CHARMM‐GUI protocol for minimization and equilibration of the systems, 500 ns production runs, with a 2 fs time step in conjunction with the LINCS algorithm^37^, were performed for each system.

To estimate the free energy landscape governing the interactions between AHM/AmB and sterols in a lipid bilayer, the metadynamics method was used^38,39^. For all systems well–tempered metadynamics runs were carried out using PLUMED 2.6.2 plugin^40^ along two reaction coordinates: distance (ξ) and angle (θ), as presented at Fig. 2B. The former coordinate was defined as the distance between the centre-of-mass (COM) of a given macrolactone ring and a sterol ring system, projected on the bilayer plane (xy-plane). The latter coordinate was defined as a torsion angle constructed by the axis of the chromophore and two vectors connecting the chromophore to the polyol chain of AHM/AmB, and the sterol ring system, projected on the xy-plane. The role of this coordinate was to trace the orientation of the sterol molecule around the antibiotic. For each studied system, 10 representative structures corresponding to ξ ≈ 10 Å and spanning the entire range of θ from 0 to 360°, with a 36° interval were chosen as starting frames for metadynamics calculations (for more detailed information, please consult Fig. S2 in Supplementary Data). Multiple walkers approach was applied with a well-tempered bias factor of 10. The history dependent bias was incremented every 5 000 steps of the simulation by depositing Gaussian-shaped repulsive potentials with a height of 0.02 kcal/mol and width of 0.005 and 0.01 kcal/mol, respectively. Reflecting walls for distance coordinate (located at 3 and 14 Å) were introduced to keep systems in the relevant region of the configurational space. All 14 simulations were conducted with 10 walkers per system. In case of AmB, for each walker an 800 ns MD trajectory was generated, whereas the convergence was reached after 700 ns (see Fig. S10). In case of AHMs, the time needed to fully probe the conformational space was longer due to the presence of an aromatic sidechain, therefore for each walker a 2 μs MD trajectory was generated. Convergence was reached after 1,7 μs (see Figs. S7, S8 and S9).

The deuterium order parameter, calculated for all 14 studied systems, clearly indicated that during all the simulations the sterol-containing DPPC bilayers were in a liquid-ordered state (Figs. S11, S12, S13 and S14).

## Supplementary Information

Supplementary Information 1.

Supplementary Information 2.

## Data Availability

Most of the data generated or analyzed during this study are included in this published article (and its Supplementary Data files). The MD trajectories generated and analyzed during the current study are available from the corresponding authors on reasonable request.
