# Supplementary material for: Antibiotic-sterol interactions provide insight into the selectivity of natural aromatic analogues of amphotericin B and their photoisomers
Source: Sci Rep. 2023 Jan 14;13:762. doi: 10.1038/s41598-023-28036-x (PMC9840637; doi:10.1038/s41598-023-28036-x)
Supplement: Supplementary file 1 — Supplementary Information 1. [file 41598_2023_28036_MOESM1_ESM.pdf]

Antibiotic-sterol interactions provide insight into the selectivity of natural aromatic analogues of amphotericin B and their photoisomers

Julia Borzyszkowska-Bukowska[1,*], Jacek Czub[2], Paweł Szczeblewski[1], and Tomasz Laskowski[1,]**

[1] Department of Pharmaceutical Technology and Biochemistry and BioTechMed Centre, Faculty of Chemistry, Gdańsk University of Technology, Gdańsk, Gabriela Narutowicza Str. 11/12, 80-233, Poland

[2] Department of Physical Chemistry, Faculty of Chemistry, Gdańsk University of Technology, Gdańsk, Gabriela Narutowicza Str. 11/12, 80-233, Poland

* julbukow@pg.edu.pl

** tomlasko@pg.edu.pl

SUPPLEMENTARY DATA

CONTENTS:

Figure S1. *Position of a sterol molecule concerning specific regions of an antibiotic molecule: A) polyene region (yellow color, angles 0-60° and 300-360°), B) in between (orange color, angles 60-120° and 240-300°) and C) polyol region (red color, angle 120-240°) with corresponding binary complex representative structures.* *3*

Figure S2. *An exemplary set 10 of starting structures for multiple-walker 2D metadynamics simulation of ParA/Chol ensemble.* *4*

Figure S3. *All representative structures of binary complexes extracted from energetic minima via cluster analysis for candicidin D.* *5*

Figure S4. *All representative structures of binary complexes extracted from energetic minima via cluster analysis for partricin A.* *6*

Figure S5. *All representative structures of binary complexes extracted from energetic minima via cluster analysis for partricin B.* *7*

Figure S6. *All representative structures of binary complexes extracted from energetic minima via cluster analysis for amphotericin B.* *8*

Figure S7. *Convergence of free energy landscapes for candicidin D: A) native with Chol, B) native with Erg, C) isomer with Chol, D) isomer with Erg.* *9*

Figure S8. *Convergence of free energy landscapes for partricin A: A) native with Chol, B) native with Erg, C) isomer with Chol, D) isomer with Erg.* *10*

Figure S9. *Convergence of free energy landscapes for partricin B: A) native with Chol, B) native with Erg, C) isomer with Chol, D) isomer with Erg.* *11*

Figure S10. *Convergence of free energy landscapes for amphotericin B: A) native with Chol, B) native with Erg.* *12*

Figure S11. *Deuterium order parameter profiles for calculated for (iso-)candicidin D/sterol/DPPC ensembles.* *13*

Figure S12. *Deuterium order parameter profiles for calculated for (iso-)partricin A/sterol/DPPC ensembles.* *14*

Figure S13. *Deuterium order parameter profiles for calculated for (iso-)partricin B/sterol/DPPC ensembles.* *15*

Figure S14. *Deuterium order parameter profiles for calculated for amphotericin B/sterol/DPPC ensembles.* *16*

Figure S15. In vitro *selective toxicity indices (STIs), calculated for all studied antibiotics, based on the data obtained for* Candida albicans *grown at two different media and reported in reference [20]. Higher numbers point to higher selective toxicity towards fungal pathogen instead of red blood cells.* *16*

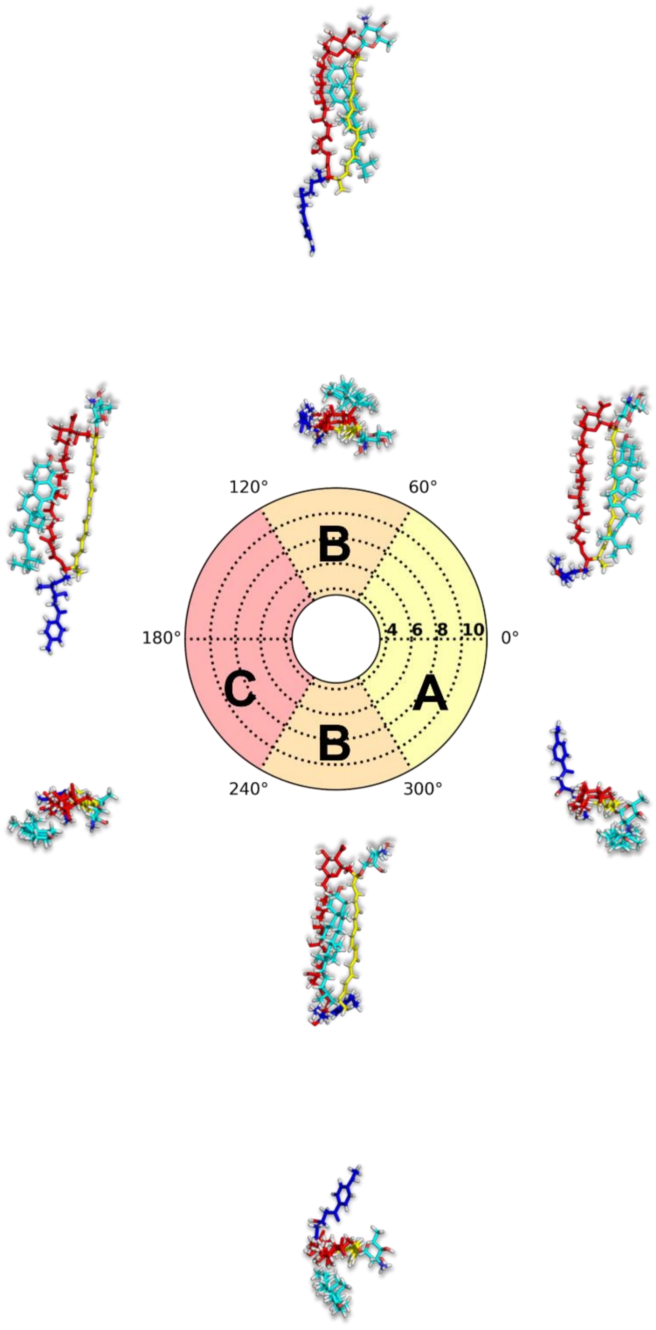

Figure S1. Position of a sterol molecule concerning specific regions of an antibiotic molecule: A) polyene region (yellow color, angles 0-60° and 300-360°), B) in between (orange color, angles 60-120° and 240-300°) and C) polyol region (red color, angle 120-240°) with corresponding binary complex representative structures.

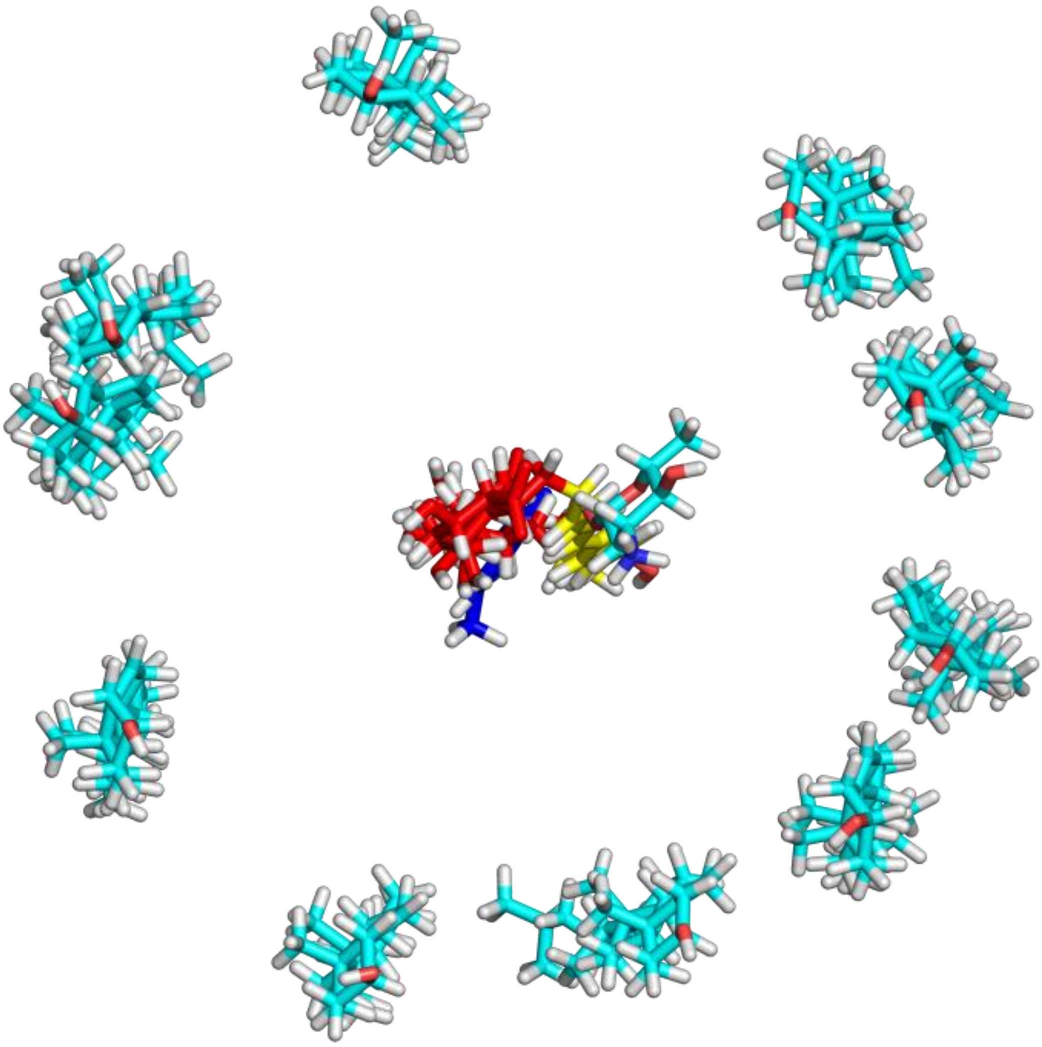

Figure S2. An exemplary set 10 of starting structures for multiple-walker 2D metadynamics simulation of ParA/Chol ensemble.

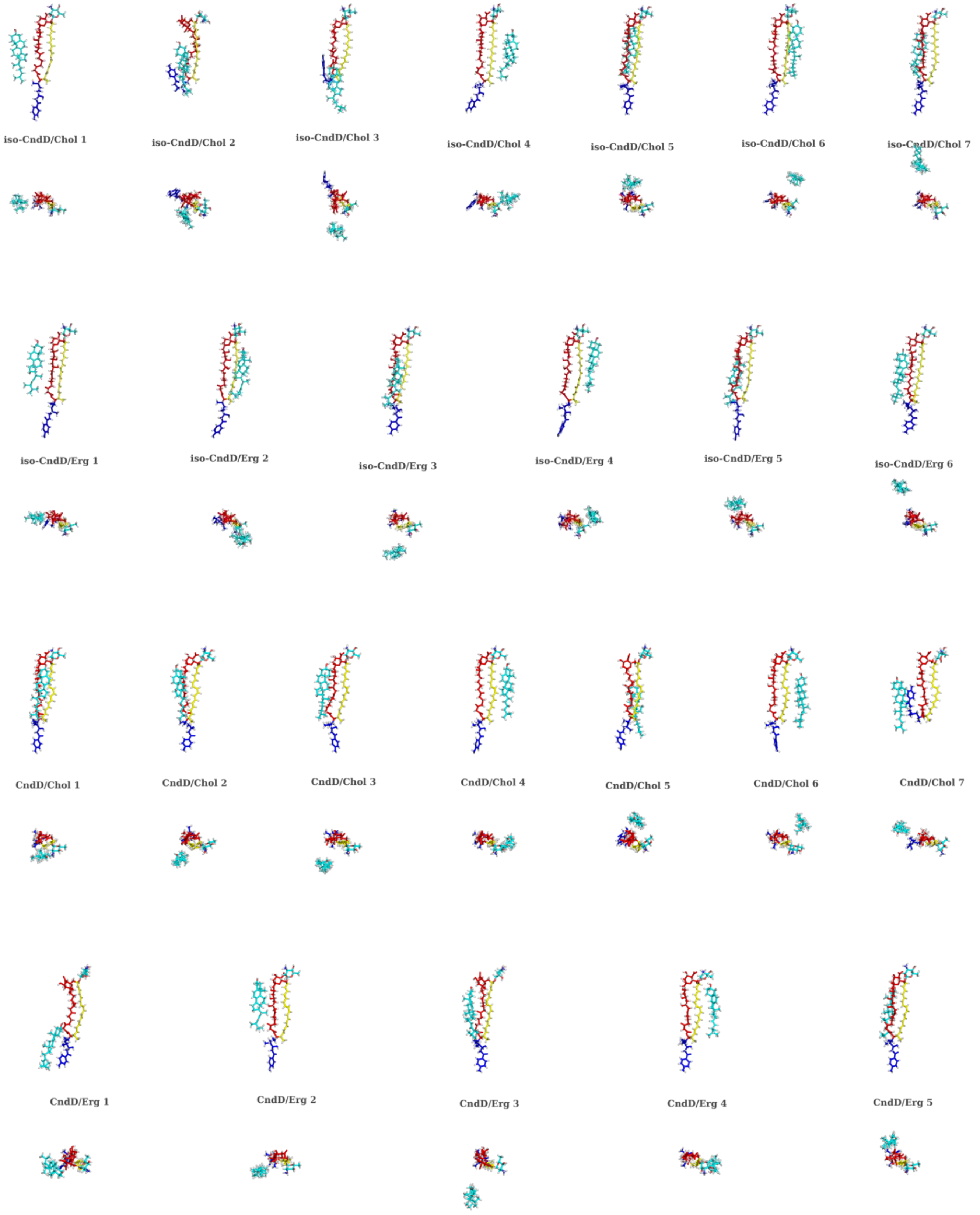

Figure S3. All representative structures of binary complexes extracted from energetic minima via cluster analysis for candicidin D.

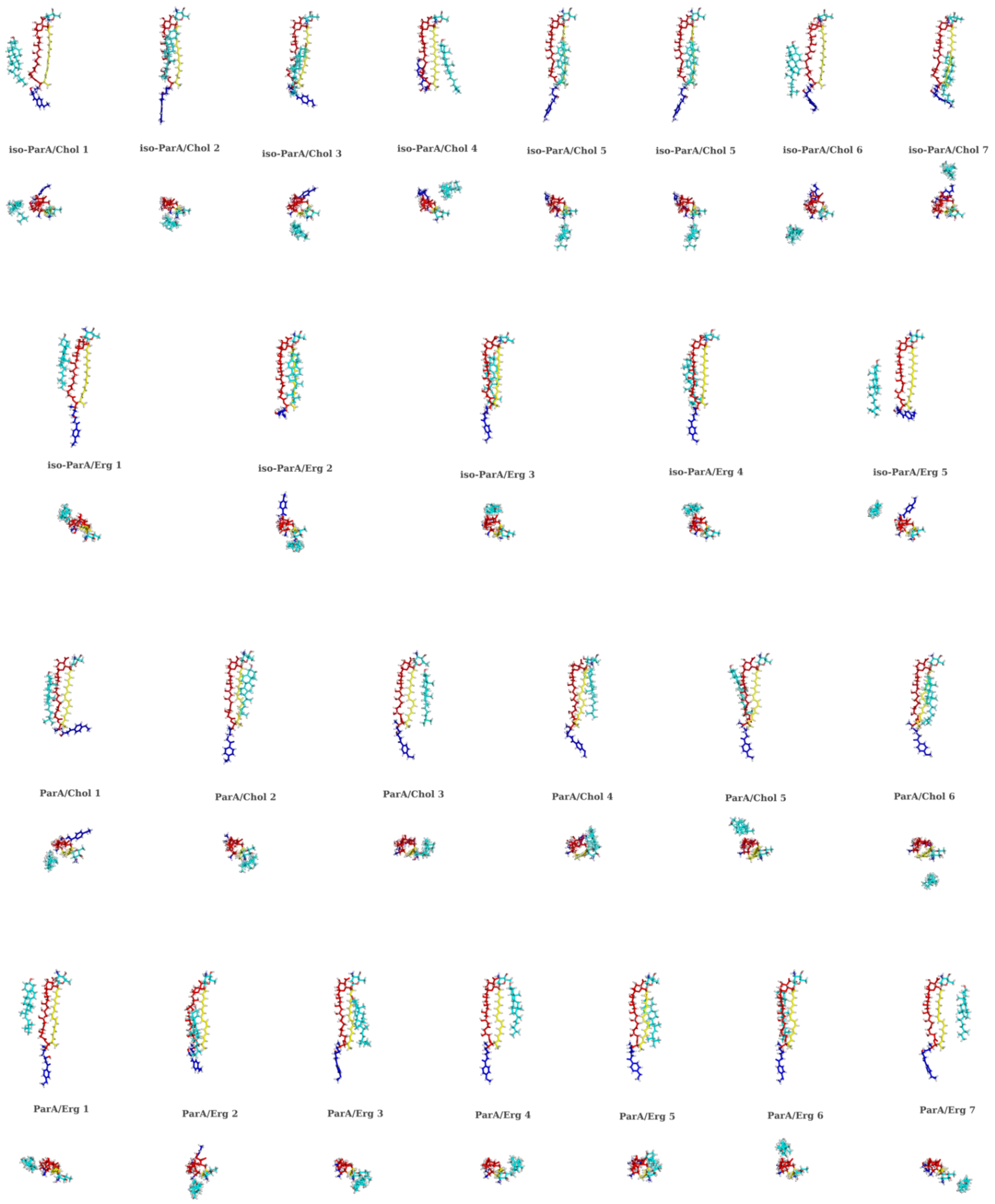

Figure S4. All representative structures of binary complexes extracted from energetic minima via cluster analysis for partricin A.

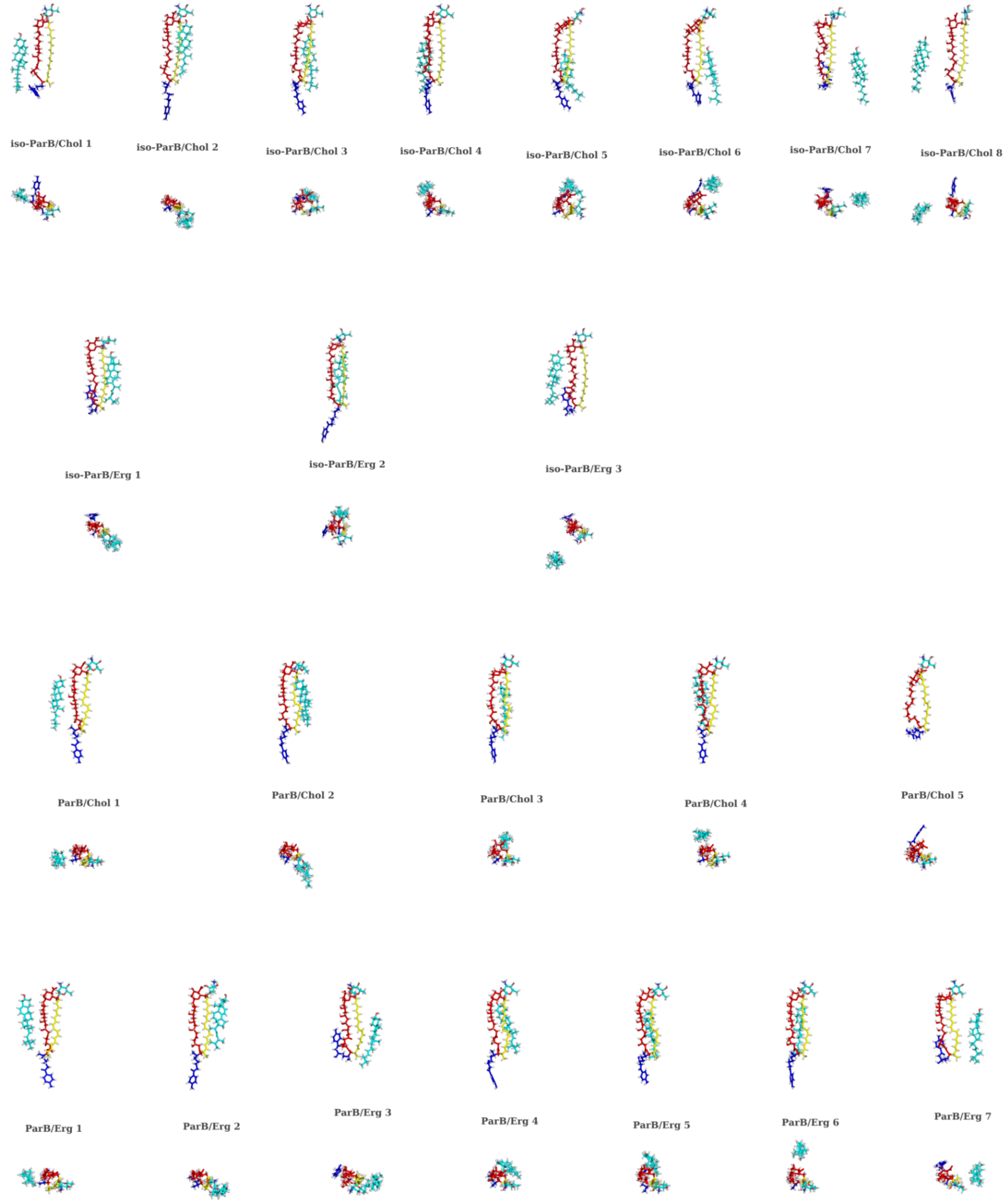

Figure S5. All representative structures of binary complexes extracted from energetic minima via cluster analysis for partricin B.

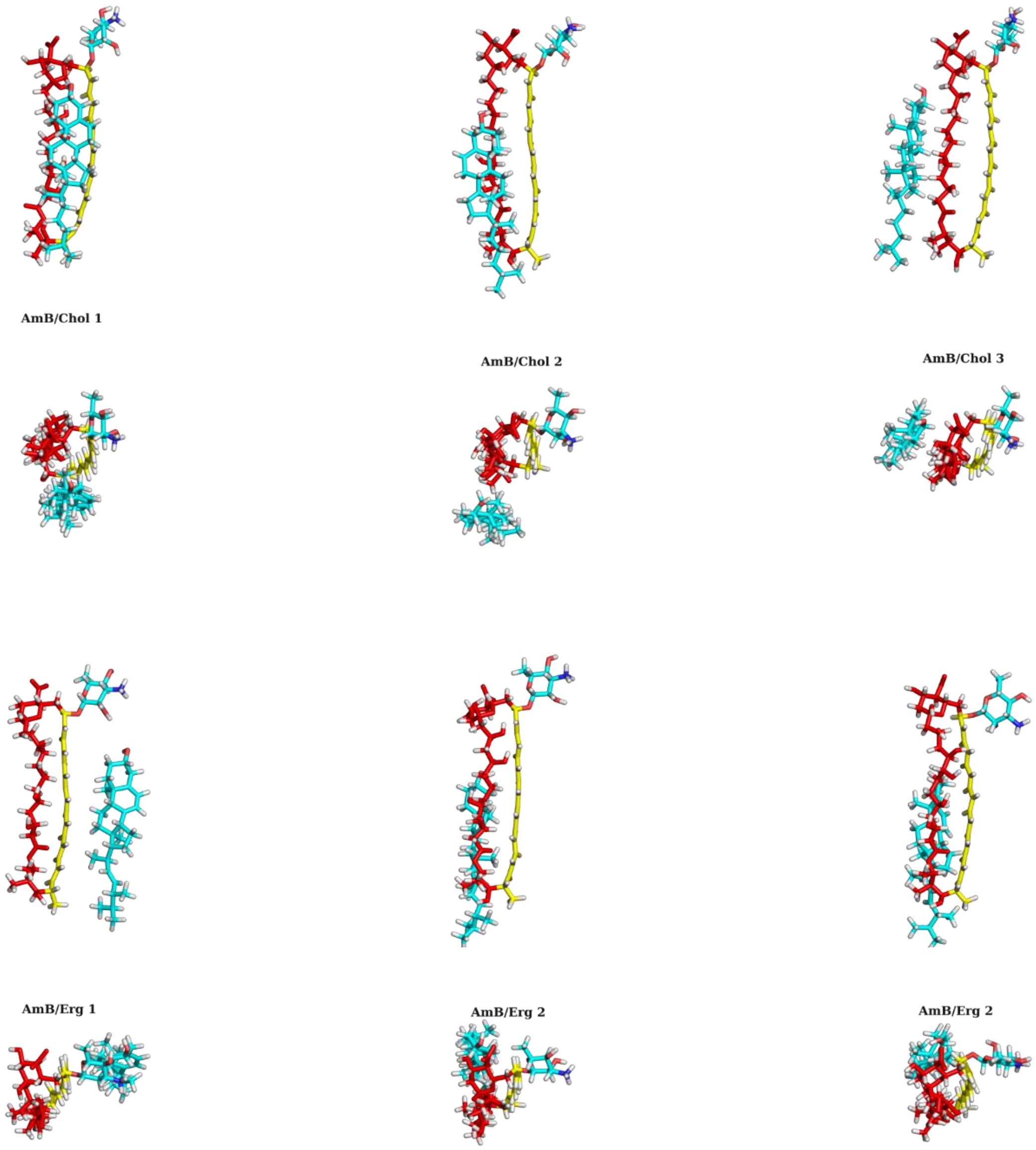

Figure S6. All representative structures of binary complexes extracted from energetic minima via cluster analysis for amphotericin B.

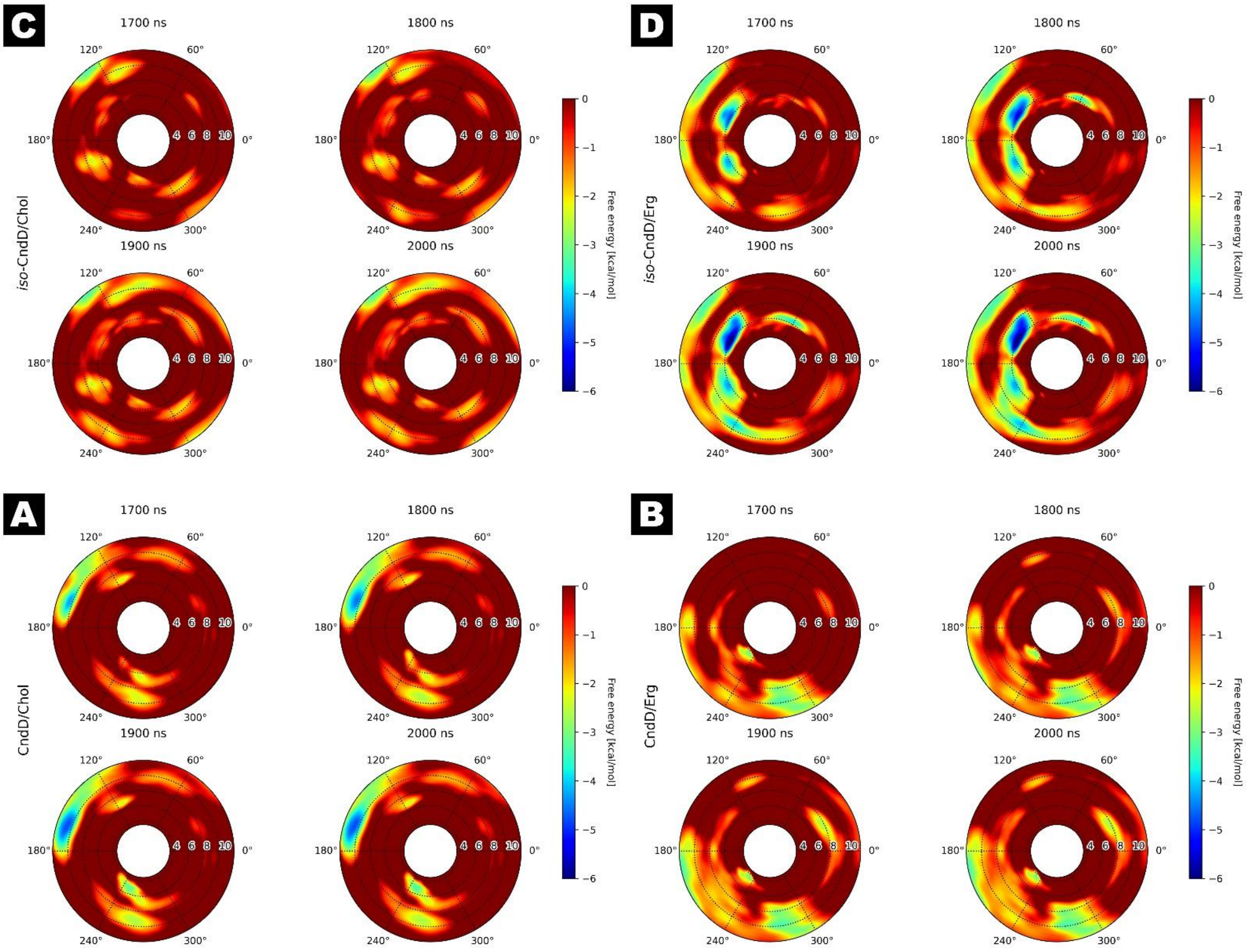

Figure S7. Convergence of free energy landscapes for candicidin D: A) native with Chol, B) native with Erg, C) isomer with Chol, D) isomer with Erg.

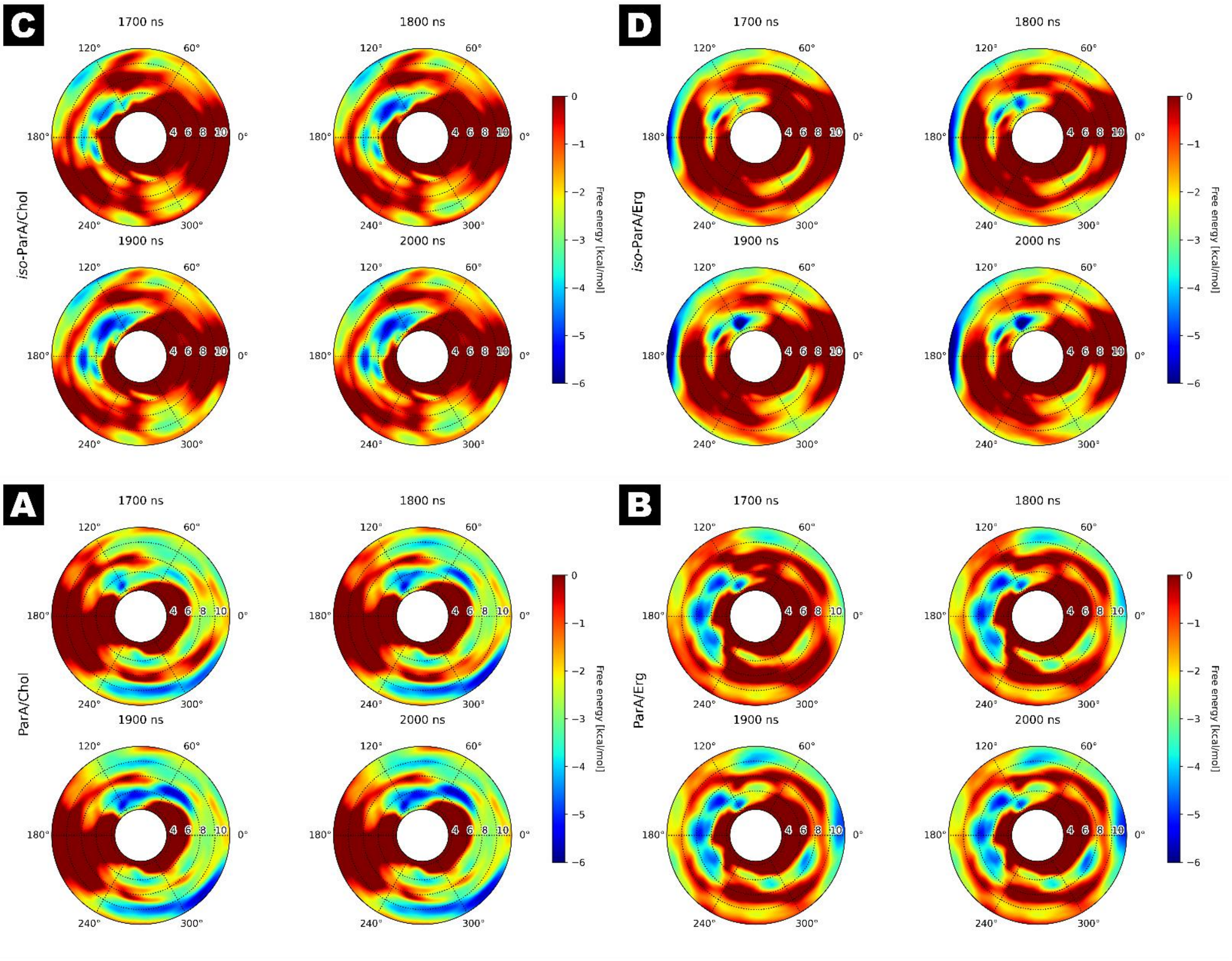

Figure S8. Convergence of free energy landscapes for partricin A: A) native with Chol, B) native with Erg, C) isomer with Chol, D) isomer with Erg.

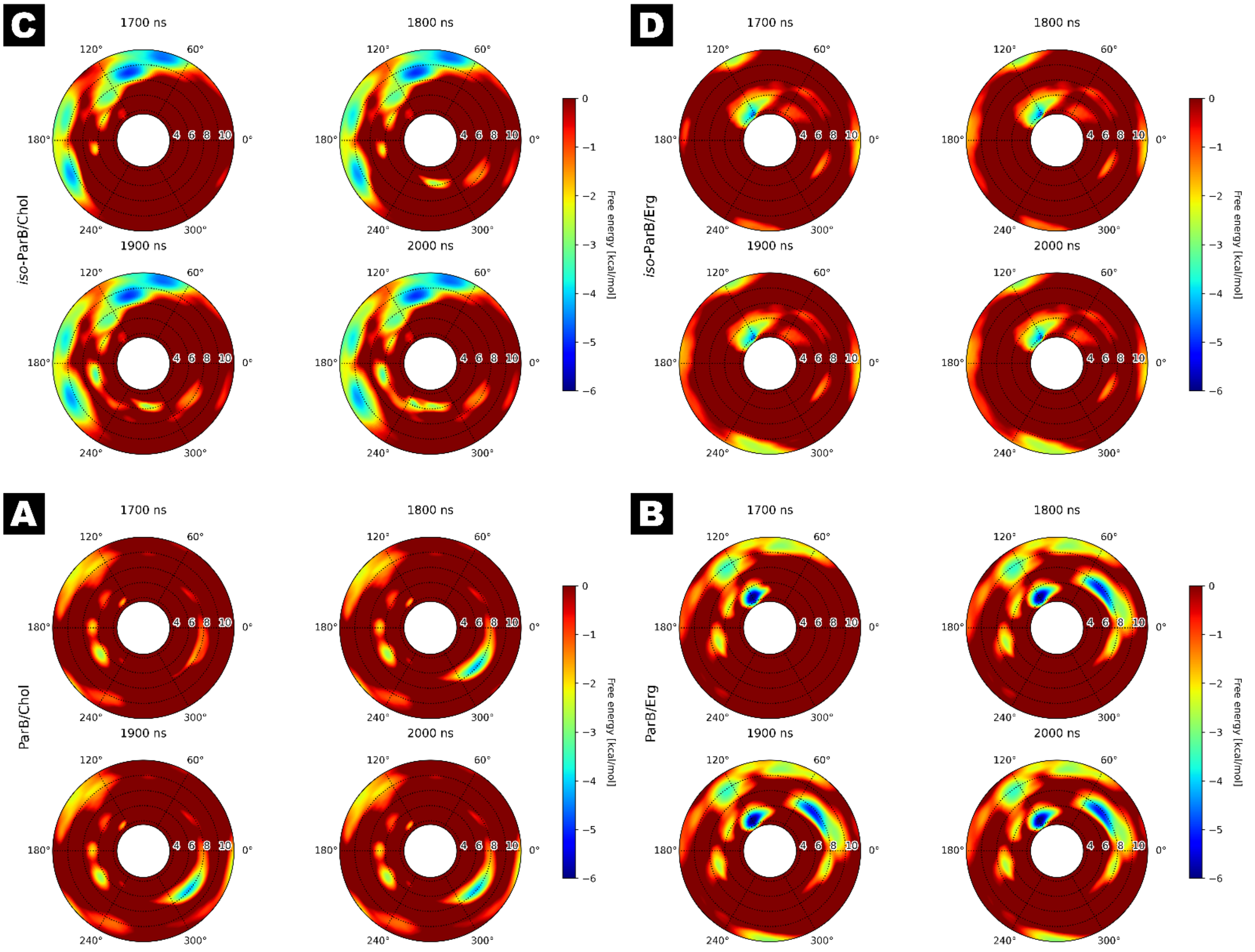

Figure S9. Convergence of free energy landscapes for partricin B: A) native with Chol, B) native with Erg, C) isomer with Chol, D) isomer with Erg.

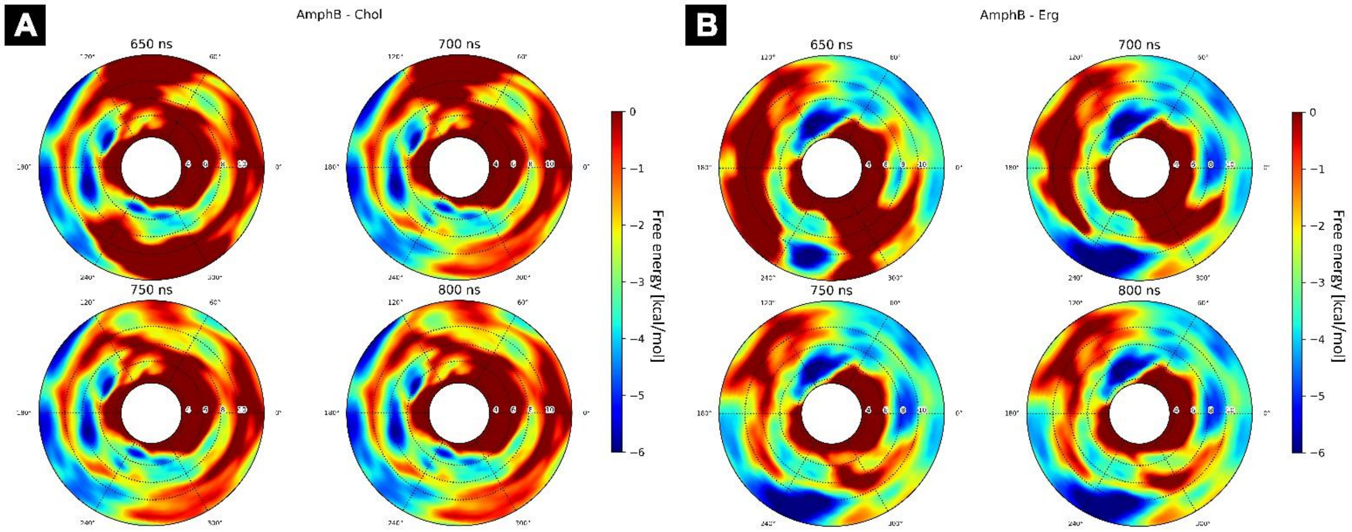

Figure S10. Convergence of free energy landscapes for amphotericin B: A) native with Chol, B) native with Erg.

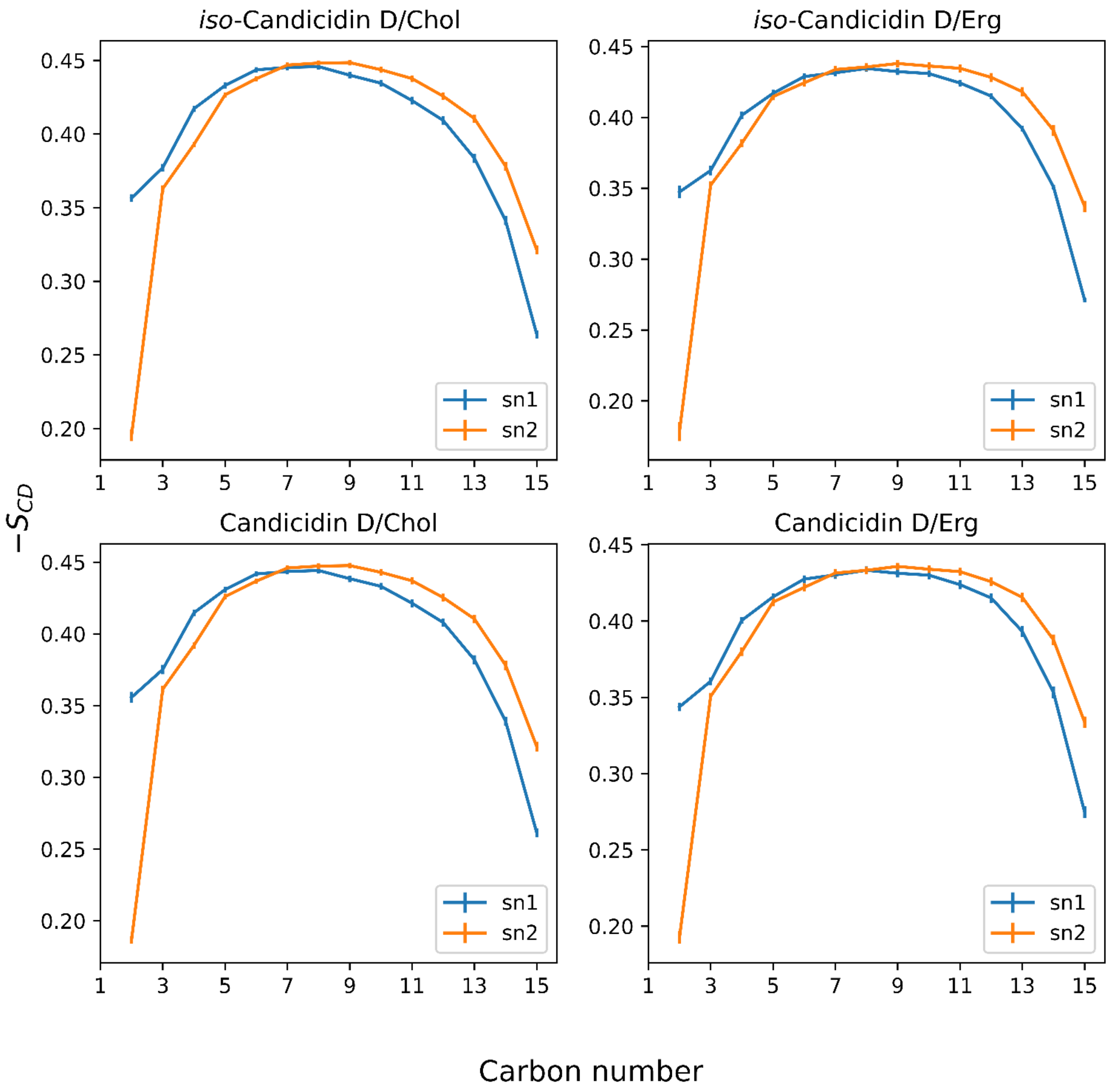

Figure S11. Deuterium order parameter profiles for calculated for (iso-)candicidin D/sterol/DPPC ensembles.

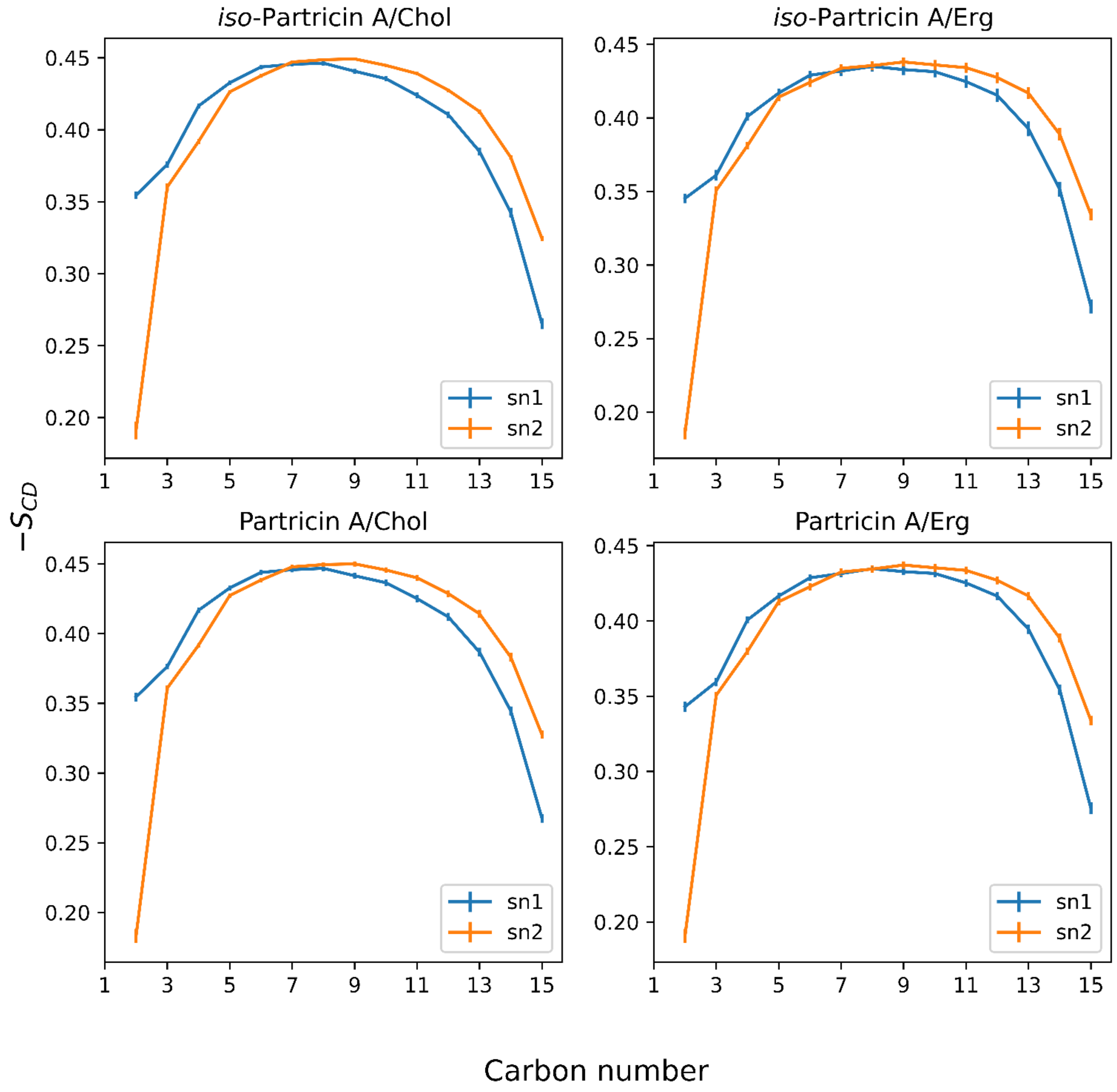

Figure S12. Deuterium order parameter profiles for calculated for (iso-)partricin A/sterol/DPPC ensembles.

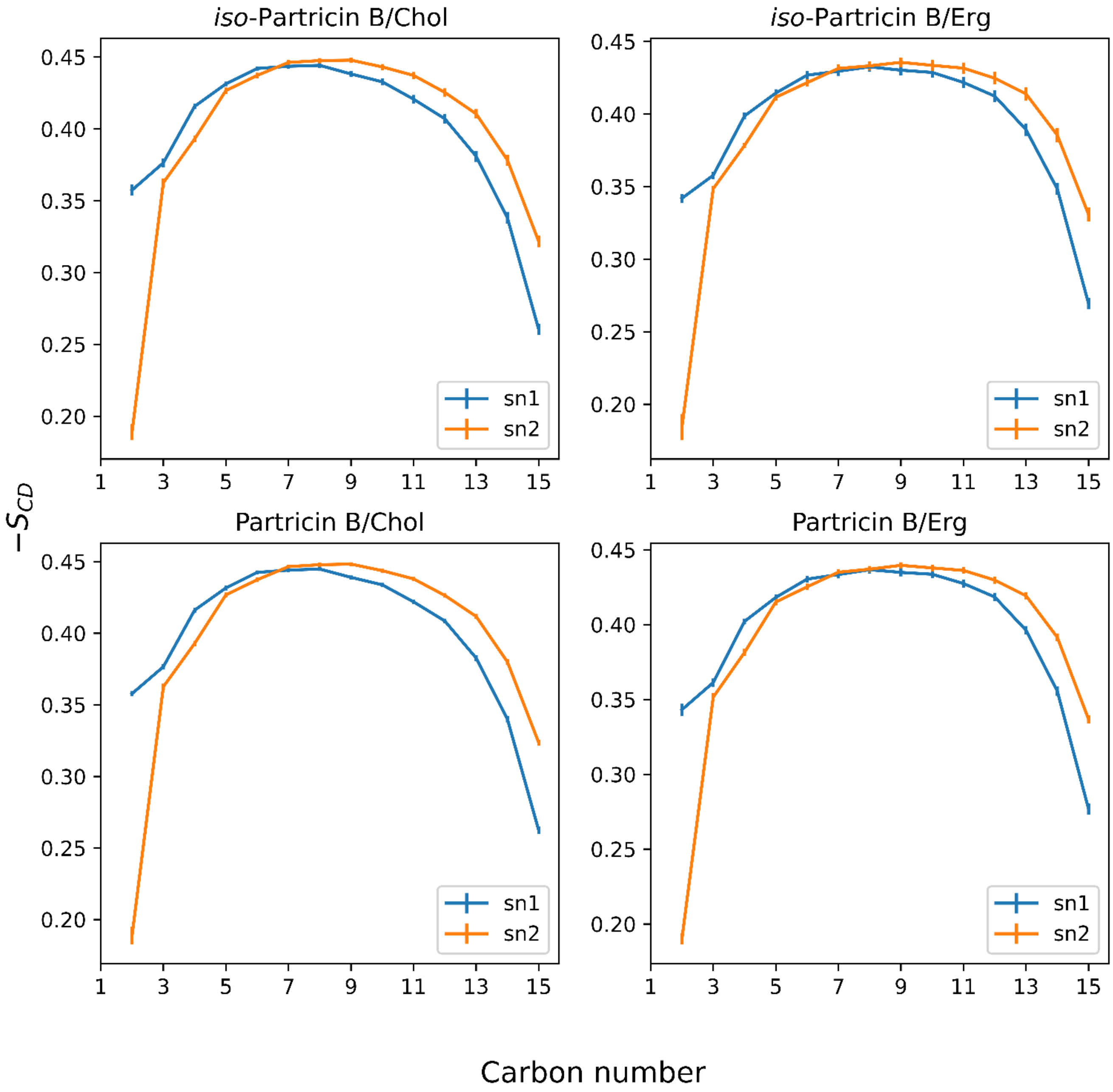

Figure S13. Deuterium order parameter profiles for calculated for (iso-)partricin B/sterol/DPPC ensembles.

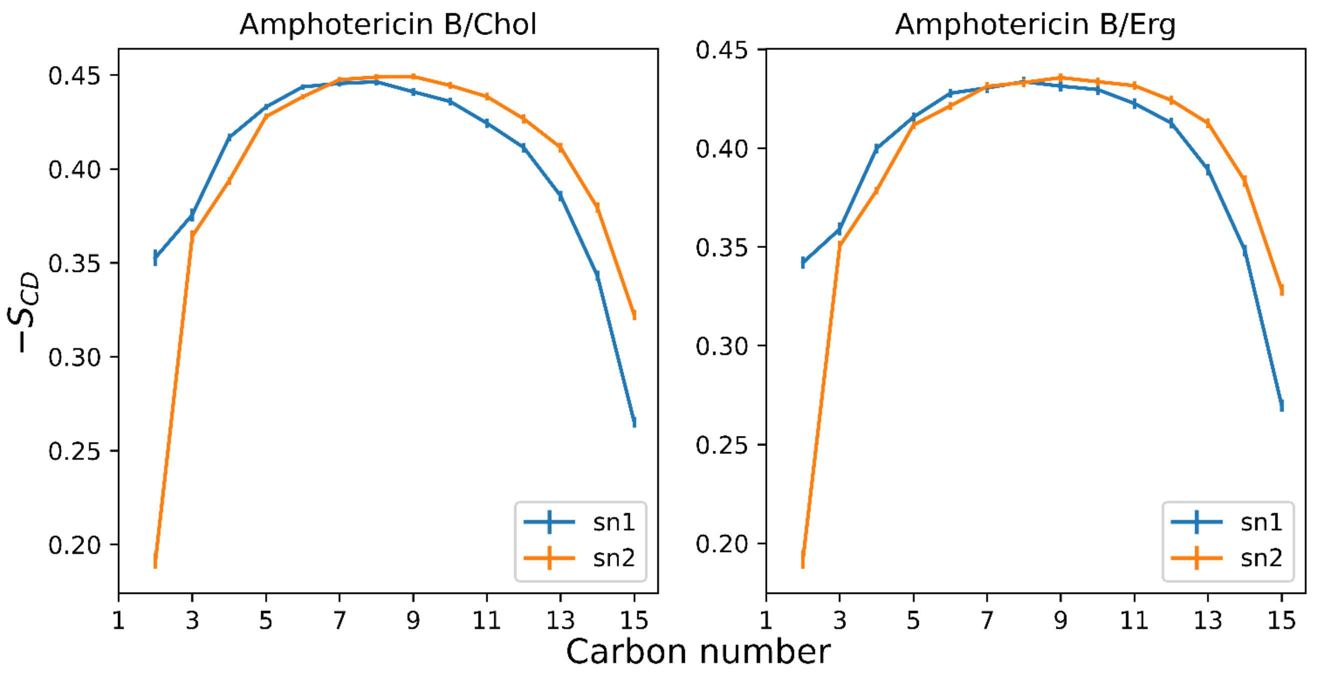

Figure S14. Deuterium order parameter profiles for calculated for amphotericin B/sterol/DPPC ensembles.

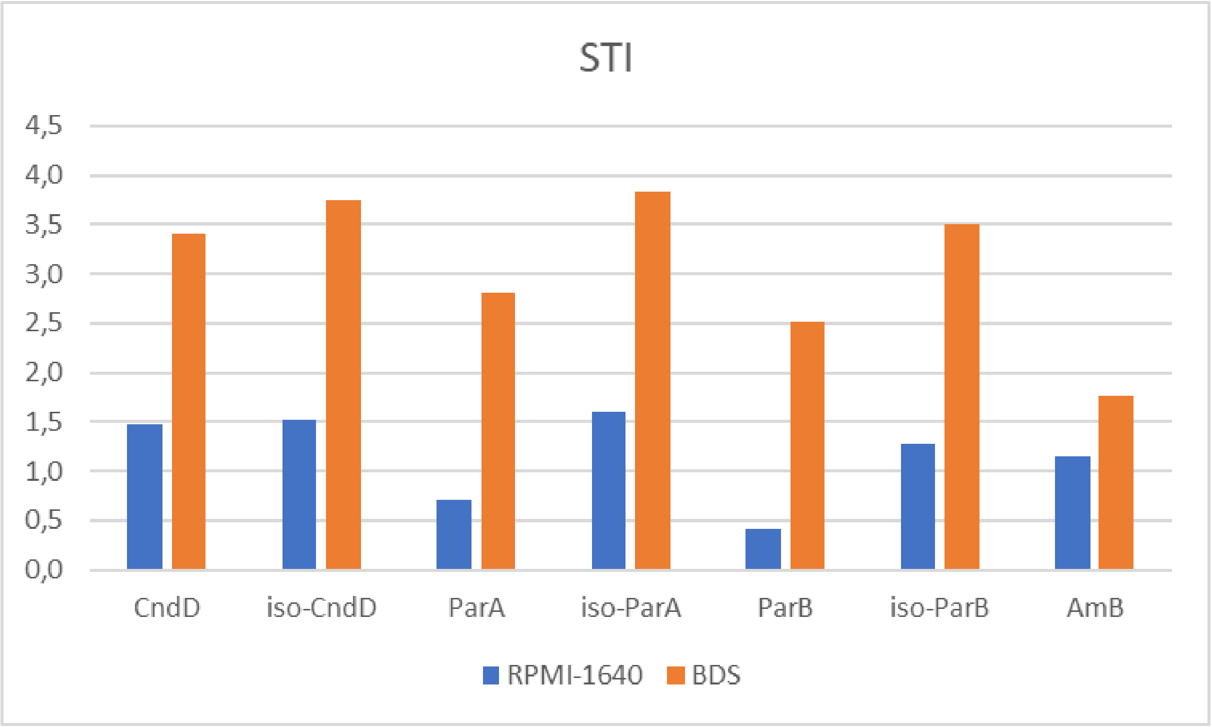

Figure S15. Decimal logarithms of *in vitro* selective toxicity indices (STIs), calculated for all studied antibiotics, based on the data obtained for *Candida albicans* grown at two different media and reported in reference [20]. Higher numbers point to higher selective toxicity towards fungal pathogen instead of red blood cells.
